# Lactic acid bacteria isolated from mammalian feces exhibit distinct diversity and probiotic traits

**DOI:** 10.1007/s11274-026-04801-8

**Published:** 2026-03-10

**Authors:** Maria Isabela da Silva Figueiredo, Ivani Souza Mello, Luana de Guimarães Bueno, Risya Regina Westphal Mendes, Jonathan Mádson dos Santos Almeida, Alan Eriksson, Gilvan Ferreira da Silva, Marcos Antônio Soares

**Affiliations:** 1 Department of Botany and Ecology, Institute of Biosciences, Laboratory of Biotechnology and Microbial Ecology, Federal University of Mato Grosso, Cuiabá, MT Brazil; 2 Department of Animal Science and Rural Extension, Faculty of Agronomy and Animal Science, Federal University of Mato Grosso, Cuiabá, MT Brazil; 3 Department of Biology and Zoology, Institute of Biosciences, Federal University of Mato Grosso, Cuiabá, MT Brazil; 4 Embrapa Western Amazon, Laboratory of Molecular Biology, Manaus, AM Brazil

**Keywords:** Caenorhabditis elegans, DAF-16/FOXO, Functional traits, Gut microbiota, Host-micromicrobe interaction, Longevity

## Abstract

Lactic acid bacteria (LAB) play an essential role in mammalian gut health and are promising candidates for probiotic use. In this study, we have identified LAB isolated from fecal samples of bats, calves, cats, or piglets or from the vaginal mucosa or teat skin of a lactating sow. Specifically, we identified 98 lineages belonging to the phyla Bacillota, Pseudomonadota, or Actinomycetota. *Enterococcus faecalis* was the only species common to all mammal groups. The LAB communities were functionally different depending on the host they originated from. *Caenorhabditis elegans* fed with *Lactiplantibacillus plantarum* PA27 lived 42.9% longer than *C. elegans* fed with *Escherichia coli* OP50. Additionally, *C. elegans* worms fed with *L. plantarum* PA17 or PA19 had longer body length than *C. elegans* worms fed with *E. coli* OP50. *L. plantarum* PA17, PA19, and PA27, isolated from piglets, were selected to evaluate how the signaling pathway (DAF-16/FOXO) was modulated during regulation of oxidative and thermal stress-related genes (*sod-3*, *hsp-16.2*, and *gcs-1*). The proportion of *C. elegans* with nuclear DAF-16 increased among worms fed with *L. plantarum* PA17, PA19, or PA27 (40% to 96%) compared to worms fed with the *E. coli* OP50 control (22%). The *sod-3* and *gcs-1* genes were more expressed in *C. elegans* fed with *L. plantarum* PA27. These results highlight that LAB isolated from mammals are potentially effective probiotics that can modulate responses to oxidative stress by activating the DAF-16/FOXO pathway, which promotes gut health and longevity.

**Supplementary Information:**

The online version contains supplementary material available at 10.1007/s11274-026-04801-8.

## Introduction

The microbiota associated with the gastrointestinal tract (GIT) represents a complex community—it is composed of different groups of microorganisms, including archaea, bacteria, fungi, viruses, and protozoa, which establish crucial relationships with the host. In mammals, the GIT-associated microbiota plays a fundamental role in promoting the host’s health (Zhang et al. 2015; Kraimi et al. 2019; Gomaa 2020): it helps nutrients to be absorbed (Judkins et al. 2020), vitamins to be synthesized (LeBlanc et al. 2013), and short-chain fatty acids to be produced (Markowiak-Kope and Slizewska 2020). Furthermore, interaction between the gastrointestinal microbiota and the immune system is crucial for maintaining homeostasis and preventing metabolic and inflammatory disorders (Lloyd-Price et al. 2019; Ruigrok et al. 2023). However, alterations in the gastrointestinal microbiota can lead to dysbiosis, thereby impacting the host’s health.

Dysbiosis, an imbalance in the gastrointestinal microbiota, occurs when the composition of microbial communities of healthy individuals’ GIT changes due to genetic factors, diet, medications, or infections by pathogenic microorganisms (Levy et al. 2017). This imbalance causes beneficial microorganism species to be lost, increases the population of harmful microbial species, or reduces microbial diversity (Petersen and Round 2014). Dysbiosis can increase intestinal permeability, promote systemic inflammation, and contribute to conditions such as metabolism-associated fatty liver disease (Alam et al. 2024). Using probiotics is a promising strategy to reverse dysbiosis.

The Food and Agriculture Organization of the United Nations (FAO) defines probiotics as “live microorganisms which, when administered in adequate amounts, confer a health benefit on the host” (FAO/WHO 2001). To be effective, probiotic candidates need to survive GIT conditions, produce bioactive substances, and adhere to intestinal surfaces to establish themselves in the host (Rokana et al. 2018; Chugh and Kamal-Eldin 2020; Naissinger da Silva et al. 2021). Probiotics promote hosts’ longevity by extending the lifespan and maintaining healthy conditions. Anti-aging effects and reduced chronic inflammation have been observed in SAMP-8 mice (an accelerated aging animal model) receiving diet supplemented with a combination of probiotic bacteria (Fang et al. 2021). Similarly, Lee et al. (2024) observed that *Lacticaseibacillus rhamnosus* IDCC 3201 increases the longevity of the nematode *Caenorhabditis elegans* by mitigating oxidative stress and immunosenescence.

The free-living nematode *C. elegans* has been widely used as an in vivo experimental model to study how probiotics promote health and longevity. This animal offers several advantages, including short life cycle, easy genetic manipulation, and digestive system that functionally resembles the digestive system of mammals (Poupet et al. 2020; Kumar et al. 2022). Moreover, mutant and transgenic *C. elegans* lineages are available, which allows specific mechanisms associated with longevity and response to stress to be explored. For example, the CF1038 lineage has a mutation in the gene encoding the DAF-2 transcription factor, which is essential for the IIS (Insulin/IGF-1 Signaling) pathway and influences longevity and response to oxidative stress in individuals (Kenyon et al. 1993; Yavorov-dayliev et al. 2022). Similarly, the OS3062 *hsp-16.2::GFP* (Bacaj and Shaham 2007) and CF1553 *sod-3p::GFP* lineages express heat shock proteins and superoxide dismutase, which are related to resistance to thermal and oxidative stress, respectively. Using *C. elegans* lineages containing GFP-tagged genes helps to elucidate the mechanisms of action of probiotics and to evaluate how probiotics influence specific stress response pathways and the well-being of organisms. These characteristics make *C. elegans* a valuable model for assessing how probiotics help to promote health and longevity.

Lactic acid bacteria (LAB) are a diverse group of Gram-positive, catalase-negative, non-spore-forming microorganisms widely recognized for their probiotic properties. In addition to producing lactic acid as their primary fermentation product (Salminen and Von Wright 2004), several LAB strains have been shown to enhance host defense and extend lifespan in *C*. *elegans* (Ikeda et al. 2007), including through the activation of skn-1, a key regulator of stress responses controlled by the p38 MAPK pathway (Komura et al. 2013, 2022). LAB include well-known genera such as *Lactobacillus* – which had their taxonomy extensively reviewed in 2020 (Zheng et al. 2020) – *Lactococcus*, *Leuconostoc*, *Streptococcus*, *Pediococcus*, *Bifidobacterium*, *Enterococcus*, and *Weissella*. Species like *Lactobacillus rhamnosus* GG^®^ (Gorbach and Goldin 1989), Lactobacillus *acidophilus* (Hansen and Mocquot 1970), *Lactobacillus plantarum* (Seddik et al. 2017), and *Bifidobacterium animalis* subsp. *lactis* BB-12^®^ (Jungersen et al. 2014) are known for their health benefits to humans and animals. Nevertheless, the functional specificity of LAB isolated from different hosts and their impact on health and longevity remain underexplored. Studies involving LAB isolated from different mammals such as bats, cats, calves, or pigs have highlighted that these bacterial communities have specific probiotic potential. The LAB isolated from bat guano in Morocco exhibit antibacterial and antioxidant activities and tolerate gastrointestinal conditions (Sakoui et al. 2022). *Lactobacillus* lineages isolated from the feces of Holstein dairy cows (*Bos taurus taurus*) in Mexico benefit essential amino acid synthesis, which functionally impacts the fecal microbiota of calves (Ruvalcaba-Gómez et al. 2023). In cats, using LAB as probiotics reduces the incidence of gastrointestinal problems and strengthens the immune system (Zha et al. 2024). Similarly, *Lactobacillus plantarum* L-27-2, *Pediococcus lactis* L-14-1, and *Enterococcus faecium* F203 obtained from fecal samples of adult Chinese cats (2 to 4 years old) show probiotic potential and hypolipidemic effects in mice (Liang et al. 2024). Additionally, *Bifidobacterium* spp and *Limosilactobacillus* spp obtained from pigs are alternatives to antibiotics for promoting growth and intestinal health in piglets and show antioxidant and antibacterial potential (Barba-Vidal et al. 2017; Dowarah et al. 2018; Dumitru et al. 2023). These findings reinforce that obtaining LAB from different origins is relevant because each isolated LAB lineage possesses specific probiotic characteristics with diverse applications.

For this study, we have hypothesized that the structure and functionality of LAB communities vary depending on the host species they originate from. Therefore, we aimed (i) to identify and to characterize the LAB lineages isolated from the feces of bats, calves, cats, or pigs; (ii) to evaluate their probiotic functional traits (resistance to gastrointestinal conditions, antimicrobial activity, and adhesion capacity), and (iii) to determine their functionalities in terms of population growth and longevity in the *C. elegans* model organism.

## Methodology

### Animals and collection of samples

Anal swab samples were collected from three adult bats (*Artibeus lituratus*) (Olfers, 1818) captured with an interception trap within the Cuiabá campus of the Federal University of Mato Grosso (UFMT) in May 2023. Anal swab samples were also collected from four calves (*Bos taurus taurus*) aged 40 days, four domestic cats (*Felis catus*) aged five days, and six piglets (*Sus scrofa domesticus*) at two time points: at the age of five and forty days. In addition, microbiota samples were collected from the mammary and vaginal regions of a lactating adult sow (*Sus scrofa domesticus*) (Table S1). After the material was collected, it was transported in a refrigerated box for processing within 3 h. The swabs were soaked in sterile saline solution (0.8% NaCl). All the collection procedures complied with animal welfare practices. Bats were captured according to the CONCEA (Conselho Nacional de Controle de Experimentação Animal, National Council for the Control of Experiments in Animals) regulations, as authorized by ICMBio (Instituto Chico Mendes de Conservação da Biodiversidade, Chicos Mendes Institute for the Conservation of Biodiversity) – SISBIO (Sistema de Autorização e Informação em Biodiversidade, System for Authorization and Information in Biodiversity) nº 75,913.

### Isolation of lactic acid bacteria (LAB)

The collected samples were serially diluted in sterile saline solution (0.8%) and plated on Man Rogosa and Sharpe (MRS) agar (Kasvi, Brazil). The plates were incubated in rigid PVC anaerobiosis jars at 30 °C for 48 h (PERMUTION, Brazil). After incubation, approximately 5 to 10 colonies with typical morphological characteristics of LAB were purified on MRS agar. The purified lineages were characterized for Gram type, cell shape, and arrangement; they were also characterized for catalase production by adding 9% (v/v) hydrogen peroxide (H_2_O_2_). The lineages were preserved in cryotubes with 20% (v/v) glycerol and stored at −80 °C for further analysis.

### Commercial probiotics

The probiotic lineages *Enterococcus faecium* NCIMB 10,415 (EF), *Lactobacillus lactis* JCM 5805 (LL), and *Lactobacillus salivarius* LS01 - DSM 22,775 (LS) were purchased from a local supplier and used as references in all the assays. The content of capsules containing a lyophilized commercial probiotic was cultured in liquid MRS medium at 30 °C for 24 h without agitation. Subsequently, the culture was diluted in saline solution (0.8%), seeded on MRS agar plates, and incubated again at 30 °C for 24 h. After incubation, typical LAB colonies were selected and used as reference in the assays.

### Identification of LAB

The isolated LAB lineages were grown in MRS broth at 30°C. After 12 h, the LAB cells were collected at 5,000 rpm for 10 min. Total DNA was extracted by using the DNA Extraction kit (Gene JET Genomic DNA Purification Kit, Thermo Scientific, USA) according to the manufacturer’s instructions. The 16S rRNA gene was amplified by PCR; the following primers were employed: forward 27F: 5’AGAGTTTGATCCTGGCTCAG3’ and reverse 1492R: 5’CCGTCAATTCCTTTGAGTTT3’ (Lane 1991). The PCR reactions were performed in a PCR thermocycler (T100 Thermal Cycler, Bio-Rad, USA) under the following conditions: initial denaturation at 94 °C for 5 min, followed by 30 denaturation cycles at 94 °C for 40 s, annealing at 55 °C for 35 s, extension at 72 °C for 1 min and 20 s, and final extension at 72 °C for 10 min. The PCR products were enzymatically purified by using ExoSap-it (GE Healthcare) and sequenced by the Sanger method, by applying BigDye Terminator Cycle Sequencing. The generated nucleotide sequences were aligned with the BioEdit software (version 7.7.1) and compared with the sequences available in the GenBank of the BLAST program (https://blast.ncbi.nlm.nih.gov/Blast.cgi). Sequences sharing 99% identity were considered to belong to the same lineage. The sequences were deposited in GenBank under accession numbers PP697637 to PP697733.

### Production of hydrolytic enzymes and phosphate solubilization capacity

Before the isolated LAB lineages were selected, secretion of hydrolytic enzymes was evaluated only in the Gram-positive and catalase-negative lineages (*n* = 68) (Adimpong et al. 2012). The hydrolytic enzymes cellulase, protease, esterase, and amylase were analyzed (Ivonilde Carrim et al. 2006). The ability to solubilize phosphate was determined in medium containing phosphate in the precipitated form (Katznelson and Bose 1959). The presence of halo, color change, or colony growth was analyzed for each methodology.

### Antibiosis against pathogenic microorganisms

Antibiosis of the isolated LAB lineages and the commercial probiotics EF, LL, and LS was determined by the agar diffusion method (Fontana et al. 2015). The following pathogenic lineages were used: the bacteria *Escherichia coli* 109U, *Staphylococcus* sp. 100U, *Klebsiella* sp. 29U, and *Pseudomonas aeruginosa* 46Ø and the yeast *Candida lusitaneae* 163, which were clinically isolated and kindly provided by a clinical analysis laboratory. Briefly, each isolated LAB lineage was cultured in MRS broth in anaerobiosis jars at 30 °C for 48 h, while each pathogen was cultured in Nutrient Broth (NB, 3 g/L meat extract, 3 g/L yeast extract, 5 g/L peptone, and 5 g/L NaCl) at 30 ° for 24 h C. After that, the optical density (OD600_nm_) of each pathogen was adjusted to 0.2 in a UV/VIS spectrophotometer, and the pathogen cells were seeded on Mueller-Hinton agar (Himedia, India) by applying the pour plate method. The medium was plated on 150 × 15 mm Petri dishes and incubated at 4 °C for 2 h until it was completely solidified. Subsequently, 6-mm-diameter wells were made in each plate, where 25 µL of a LAB culture or an antibiotic (positive control) was placed. The positive control was ampicillin (100 mg/mL), tetracycline (100 mg/mL), or chloramphenicol (50 mg/mL). The plates were then incubated at 30 °C for 24 h. The presence of inhibition halos around the well indicated positive antibiosis. Data are expressed as diameter (mm) of the inhibition halos.

### Cell surface properties

The isolated LAB lineages that showed antimicrobial activity against at least one of the tested pathogens were selected (*n* = 26). Each selected lineage was cultured in MRS broth in anaerobiosis jars at 30 °C for 48 h, while each pathogen was cultured in NB at 100 rpm and 30 °C for 24 h. The suspension was collected by centrifugation at 5000 rpm for 10 min, rinsed twice in 0.05 M Phosphate Buffered Saline (PBS), pH 6.8, and resuspended in the same buffer.

### Cell surface hydrophobicity assay

The surface hydrophobicity of the selected LAB lineages was determined according to (Ekmekci et al. 2009)et al. (2009). The affinity of the lineages for xylene was quantified in a two-phase system. A suspension with OD600_nm_ of 0.2 was prepared for each lineage. Subsequently, 1 mL of xylene was added to a test tube containing 3 mL of a selected lineage suspension. The tube was mixed on a vortex for 90 s and left to rest for 30 min, to allow the two phases to separate. The aqueous phase was collected, and OD600_nm_ was quantified. Hydrophobicity was calculated from three replicates, by considering the decrease in the percentage of the optical density of the original lineage suspension due to partitioning of the LAB cells in the hydrocarbon layer. The percentage of cell surface hydrophobicity was calculated by using the following equation:

$$\:\mathrm{H}\mathrm{y}\mathrm{d}\mathrm{r}\mathrm{o}\mathrm{p}\mathrm{h}\mathrm{o}\mathrm{b}\mathrm{i}\mathrm{c}\mathrm{i}\mathrm{t}\mathrm{y}\:\left(\mathrm{\%}\right)=\left(\frac{\left[\mathrm{O}\mathrm{D}600\:\mathrm{b}\mathrm{e}\mathrm{f}\mathrm{o}\mathrm{r}\mathrm{e}\:\mathrm{m}\mathrm{i}\mathrm{x}\mathrm{i}\mathrm{n}\mathrm{g}-\:\mathrm{O}\mathrm{D}600\:\mathrm{a}\mathrm{f}\mathrm{t}\mathrm{e}\mathrm{r}\:\mathrm{m}\mathrm{i}\mathrm{x}\mathrm{i}\mathrm{n}\mathrm{g}\right]}{\mathrm{O}\mathrm{D}600\:\mathrm{b}\mathrm{e}\mathrm{f}\mathrm{o}\mathrm{r}\mathrm{e}\:\mathrm{m}\mathrm{i}\mathrm{x}\mathrm{i}\mathrm{n}\mathrm{g}}\right)\:\mathrm{X}\:100$$

### Self-aggregation assay

Self-aggregation of the selected LAB lineages was measured according to the method described by Jena et al. (2013), with some modifications. Each lineage suspension (OD600_nm_ = 0.2) was incubated at 30 °C for 24 h. After incubation, the OD was re-evaluated. The self-aggregation percentage was determined according to the following equation:

$$\:\mathrm{S}\mathrm{e}\mathrm{l}\mathrm{f}-\mathrm{a}\mathrm{g}\mathrm{g}\mathrm{r}\mathrm{e}\mathrm{g}\mathrm{a}\mathrm{t}\mathrm{i}\mathrm{o}\mathrm{n}\:\left(\mathrm{\%}\right)=1-\left(\frac{\mathrm{A}24\mathrm{h}}{\mathrm{A}0\mathrm{h}}\right)\mathrm{X}\:100$$

where A24h represents the OD600_nm_ at 24 h and A0 is the OD600_nm_ at time = 0.

### Co-aggregation assay with pathogens

Co-aggregation of each selected LAB lineage with a pathogen *(E. coli* 109U, *Staphylococcus* sp. 100U, *Klebsiella* sp. 29U, *P.* aeruginosa 46Ø, or *C.* lusitaneae 163) was evaluated. Each lineage suspension was grown in MRS broth in anaerobiosis jars at 30 °C for 48 h, and each pathogen was grown in NB at 30 °C for 24 h. The OD600_nm_ was adjusted to 0.2. On a vortex, a lineage suspension (2 mL) was mixed with 2 mL of one of the pathogen suspensions for at least 10 s. To this end, 200 µL of a lineage + pathogen suspension was added to a 96-well plate and incubated in anaerobiosis jars at 30 °C for 24 h. The co-aggregation rate is expressed as follows:

$$\:\mathrm{C}\mathrm{o}-\mathrm{a}\mathrm{g}\mathrm{g}\mathrm{r}\mathrm{e}\mathrm{g}\mathrm{a}\mathrm{t}\mathrm{i}\mathrm{o}\mathrm{n}\:\left(\mathrm{\%}\right)=100\:X\:\left(Amix24h-Amix0h\right)$$

where Amix0h represents the OD600_nm_ of a LAB + pathogen mixture at t = 0, and Amix24h represents the OD600_nm_ of a LAB + pathogen mixture after incubation for 24 h.

### Tolerance to GIT conditions

The tolerance of each selected LAB lineage or commercial probiotic to pH 2.5 and 0.3% bile was evaluated as described by (Huang et al. 2020), with modifications. Each lineage was cultured in MRS broth for 48 h, and the optical density was adjusted to OD600_nm_ = 0.2. For the pH tolerance test, 200 µL of a lineage suspension was added to a tube containing 5 mL of MRS at pH 2.5. For the bile tolerance test, the tube contained MRS supplemented with 0.3% bacteriological bovine bile (Oxgall, Biolog). In both assays, a 20 µL aliquot of lineage or commercial probiotic culture was immediately plated on MRS agar (time 0) and incubated in anaerobiosis jars at 30 °C for 2 h (pH tolerance test) or 8 h (bile tolerance test). Tolerance was determined on the basis of viable colony count (in triplicate) and reflected survival to gastric and intestinal transit time (pH and bile tolerance test, respectively).

### Safety assessment

Relevant characteristics for probiotic safety, such as resistance to antibiotics and hemolytic activity, were used to evaluate the safety of each selected LAB lineage and commercial probiotic (Lahtinen et al. 2009).

### Susceptibility to antibiotics

The susceptibility of each selected LAB lineage to antibiotics was evaluated according to Angmo et al. (2016), with modifications. Each lineage or commercial probiotic was grown in MRS broth in anaerobiosis jars at 30 °C for 48 h. After incubation, Petri dishes (150 × 15 mm diameter) containing Mueller-Hinton agar medium (Himedia, India) were seeded with 100 µL of a lineage or commercial probiotic suspension. Paper discs containing the antibiotics piperacillin + tazobactam (110 µg), meropenem (10 µg), penicillin (10 IU), imipenem (10 µg), ceftazidime (30 µg), gentamicin (10 µg), polymyxin b (300 IU), oxacillin (1 µg), cefoxitin (30 µg), cefepime (30 µg), ciprofloxacin (5 µg), and sulfazotim (25 µg) were deposited on the medium surface. After incubation at 30 °C for 24 h, the diameter of the inhibition halos was measured in millimeters, and the lineages were categorized as sensitive (≥ 21 mm), intermediate (16–20 mm), or resistant (≤ 15 mm) (Reuben et al. 2019).

### Hemolytic activity

The selected LAB lineages were streaked on the surface of tryptic soy agar (TSA) plates (Newprov, Brazil) supplemented with 50 mL/L sheep blood. After incubation at 30 °C for 48 h, the plates were examined for β-hemolysis (clear zones around colonies), α-hemolysis (green zones around colonies), or γ-hemolysis (absence of zones around colonies). The *Staphylococcus* sp. 92 S lineage, kindly provided by the Veterinary Hospital of the Federal University of Mato Grosso, was used as positive control.

#### *In vivo* probiotic potential

The LAB lineages (*n* = 6) displaying more promising probiotic characteristics such as antimicrobial activity against four or more evaluated microorganisms, tolerance to pH (2.5) and bile (0.3%), and high rates of hydrophobicity, self-aggregation, and co-aggregation with pathogens (above 50%) were selected for in vivo probiotic potential evaluation.

#### *Caenorhabditis elegans *lineages and culture

The *C*. *elegans* lineages N2 (wild type), BA17 [*fem-1*(*hc17*) *IV*], CF1553 [*muIs84 (sod-3p::GFP*)], LD1171 [*ldIs7 (gcs-1p::GFP*)], CL2070 [*dvIs70 (hsp-16.2p::GFP*)], and TJ356 [*zIs356 (daf-16p::daf-16a/b::GFP + rol-6(su1006*)] were maintained at 15 °C on plates containing nematode growth medium (NGM) (2.5 g/L peptone, 3 g/L NaCl, 15 g/L bacteriological agar, 1 M MgSO4·7H_2_O, 1 M CaCl_2_, 5 mg/mL cholesterol, and 1 M KPO_4_). *E. coli* OP50, previously cultured in Luria-Bertani (LB) broth (10 g/L peptone, 5 g/L yeast extract, and 5 g/L NaCl), was used as food.

### Longevity assay with C. elegans BA17

The mutant lineage *C. elegans* BA17 is temperature-sensitive, which results in the population being feminized at 25 °C, thereby preventing offspring from being produced. The use of *C. elegans* BA17 eliminates the need for FUdR (5-fluoro-2’-deoxyuridine) used in experiments with the wild-type *C. elegans* N2. The use of this nucleoside analog should be avoided to prevent false positives or false negatives (Saul et al. 2021). This lineage was used to evaluate whether any of the selected LAB lineages contributes to extending the lifespan of nematodes without interfering in progeny (De Bonomo et al. 2014; Peixoto et al. 2017, 2018, 2019). The longevity assay was performed in liquid medium (Solis and Petrascheck 2011; Zwirchmayr et al. 2020; Kumaree et al. 2023; Carrara et al. 2024) according to the method described by Kumaree et al. (2023), with modifications. Briefly, the lifespan was evaluated in 96-well plates containing K liquid medium (2.36 g/L KCl, 3 g/L NaCl) (Boyd et al. 2012). *E. coli* OP50 (negative control), or one of the commercial probiotics LL, LS, or EF (positive controls) were previously grown in MRS broth or LB broth (for *E. coli* OP50) and maintained in an incubator at 30 °C for 48 h. After that, the cultures were washed with 0.8% saline solution, and 200 µL of individual cultures with OD600_nm_ of 1.0 were added to the wells. Approximately 20 synchronized L4 stage *C. elegans* BA17 were transferred to wells, and the worm survival was recorded every two days. Worms that did not move after being touched were considered dead. The assay was performed in triplicate for each selected LAB lineage or control.

### Reproduction and body length assay with C. elegans N2

Each selected LAB lineage was cultured in MRS broth; *E. coli* OP50 was cultured in LB; and the cultures were incubated at 30 °C for 48 h. Then, each culture was centrifuged at 5000 rpm for 10 min and rinsed twice with 0.8% saline solution, and its OD600_nm_ was adjusted to 1.0. Subsequently, 300 µL of one of the resulting suspensions was transferred to a Petri dish (75 mm) containing NGM and incubated at 30 °C for 48 h. Afterwards, approximately 20 synchronized L4 stage worms were added to each plate and incubated at 20 °C for 10 days. Then, the worms were removed and rinsed into K medium for the population to be evaluated. Aliquots of known volumes were observed under a microscope, and young and adult worms were counted. The assay was performed at least three times for each treatment and control.

The body length was determined for approximately 20 adult worms immobilized in 2% agarose gel prepared with 20 mM sodium azide. The worms were visualized and photographed with an Olympus SZ61 microscope by using a 10x objective lens. The photographs were used to evaluate the body length of the worms by using the ImageJ software (version 1.54d) (Schneider et al. 2012).

### Evaluation of the activation of the antioxidant and thermal stress pathways in C. elegans

Each selected LAB lineage was cultured in MRS broth (25 mL) at 30 °C for 48 h. Then, each culture was centrifuged at 5000 rpm for 10 min, washed three times with sterile 0.8% saline solution, and resuspended in the same solution. Subsequently, 300 µL of a suspension was plated on Petri dishes (75 mm) containing NGM and incubated at 30 °C for 48 h.

The following *C. elegans* lineages were used: CF1553 [*muIs84* (*sod-3p::GFP*)], LD1171 [*ldIs7* (*gcs-1p::GFP*)], and CL2070 [*dvIs70* (*hsp-16.2p::GFP*)]. Each nematode was maintained on NGM plates with *E. coli* OP50 and synchronized by alkaline lysis, and the resulting eggs were plated directly on plates containing a LAB lineage or *E. coli* OP50 (control). The plates were incubated at 20 °C for 24 h. After that, the nematodes were washed with sterile M9 buffer (M9, 3 g/L KH_2_PO_4_, 6 g/L Na_2_HPO_4_, 5 g/L NaCl, and 1 M MgSO) to remove excess bacteria and then mounted on microscopy slides. For immobilization, 50 µL of suspension with treated worms was transferred to slides containing 50 µL of 20 mM sodium azide. The preparations were covered with coverslips and observed under a fluorescence microscope equipped with a green channel filter. The images were obtained under 20x magnification and subsequently analyzed by using the ImageJ software (version 1.54d) (Schneider et al. 2012). The fluorescence intensity was measured in standardized regions of interest.

### DAF-16 translocation in C. elegans exposed to LAB lineages with probiotic potential

The mutant *C. elegans* lineage TJ356 [*zIs356 (daf-16p::daf-16a/b::GFP + rol-6(su1006*)] was used to evaluate the subcellular translocation of the DAF-16 transcription factor. The nematodes were synchronized by lysis with alkaline solution and were then cultured with *E. coli* OP50 on NGM plates at 20 °C until they reached the L4 stage. This stage was chosen to avoid early mortality caused by some of the LAB lineages. After the L4 stage was reached, the nematodes were washed with sterile M9 buffer to remove excess OP50, and then approximately 1000 worms were transferred to Petri dishes (75 mm) containing NGM seeded with a selected isolated LAB lineage with probiotic potential. The nematodes were exposed to a selected LAB lineage or the *E. coli* OP50 control at 20 °C for 48 h. Next, the nematodes were washed with sterile M9 to remove excess bacteria and divided into groups of approximately 300 worms per 1.5-mL microtube. The nematodes fed with a selected LAB lineage or the *E*. *coli* OP50 control were subdivided into two conditions: (i) no stress (control), 20 °C; and (ii) oxidative stress, exposure to 5 mM hydrogen peroxide (H₂O₂) for 2 h. After exposure to a certain condition, the nematodes were anesthetized with 20 mM sodium azide and immediately mounted on microscopy slides for fluorescence analysis. The DAF-16 subcellular localization was observed under a fluorescence microscope and classified into three distinct patterns: cytoplasmic, nuclear, or intermediate. For each experimental condition, 50 to 80 nematodes were evaluated.

### Statistical analysis

Differences between treatments were estimated by one-way analysis of variance (ANOVA) or Student’s t-test. When necessary, Dunnett’s post hoc test was used to verify comparisons of treatments with the control, and Tukey’s post hoc test was used for multiple comparisons. Analyses were performed with the R software (R Core Team (2023) by using the standard stats package and the multcompView package (Graves et al. 2015). Survival curves and statistical differences were analyzed by the log-rank (Mantel-Cox) test; the GraphPad Prism software, version 10.2.3 for Windows (GraphPad Software, San Diego, CA, USA), was employed. The degree of similarity between different communities was determined on the basis of the Jaccard index (Hancock 2004), while UPGMA (Unweighted Pair Group Method with Arithmetic Mean) cluster analysis grouped communities into distinct clusters. Multivariate Principal Component Analysis (PCA) was used to verify differences in the composition of bacterial communities regarding functional traits as compared to standardized data, and differences were confirmed through permutation analysis by using the PERMANOVA test and the Past software (version 4.15) (Dasgupta 2013).

## Results

### Bacterial communities isolated from healthy mammals

**Fig. 1:**
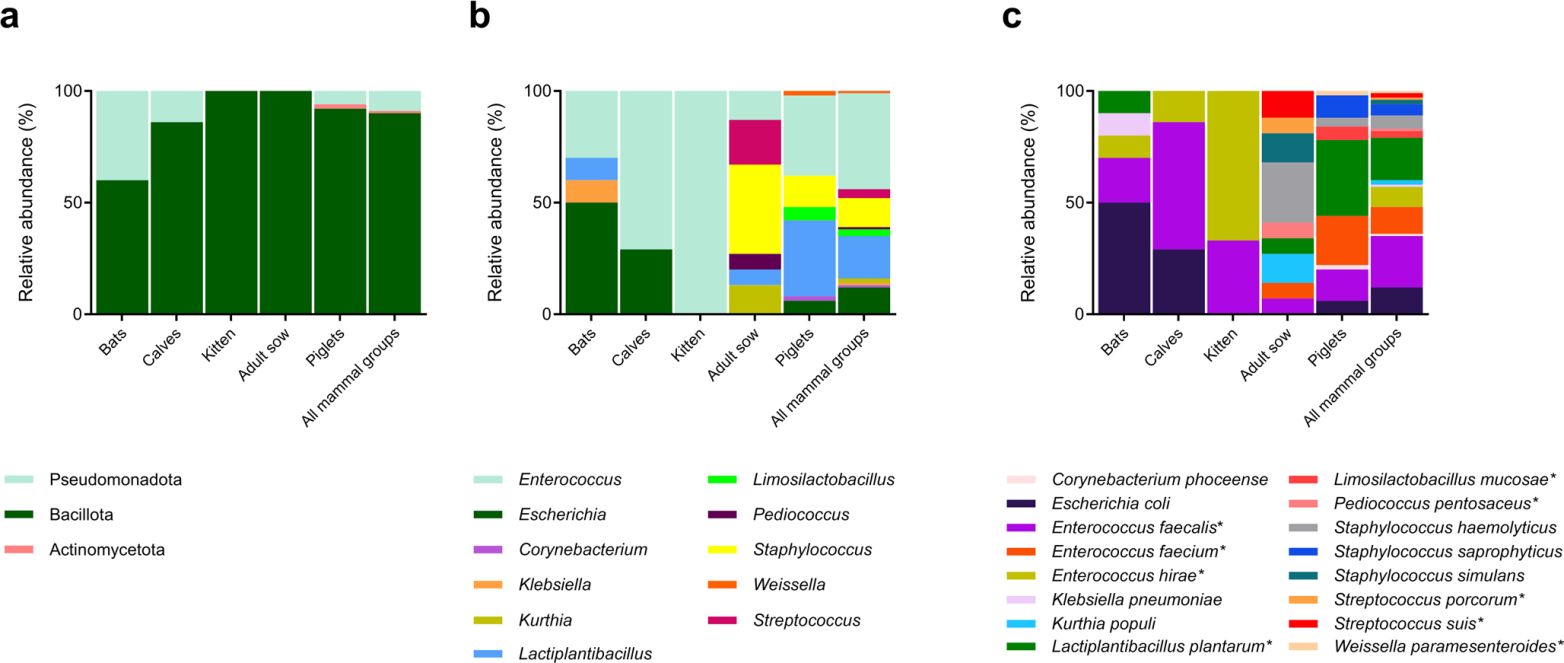
Relative abundance (%) of phyla (**a**), genera (**b**), and species (**c**) present in samples obtained from different mammalian species, as determined on the basis of 16S rRNA.* Indicates lactic acid bacteria lineages

A total of 27 samples were analyzed. The samples comprised fecal samples obtained from bats, calves, or piglets or samples obtained from the vaginal region or teat skin of a lactating adult sow (Table S1). We obtained 111 bacterial lineages, 13 of which did not remain viable. Therefore, we molecularly identified the remaining 98 lineages by partial sequencing of the 16 S rDNA gene (Table S1), which revealed that the isolated lineages belonged to the phyla Bacillota (90%), Pseudomonadota (9%), or Actinomycetota (1%) (Fig. 1a). We detected Actinomycetota, the least abundant phylum, in piglets only. The strains were distributed across 11 genera (Fig. 1b) and 16 species (Fig. 1c), of which 6 genera and 8 species are LAB. *Enterococcus faecalis* was the most abundant species (21%) and the only one shared by all the evaluated mammalian groups (Fig. 1c). Of the 98 identified lineages, 68 were Gram-positive and catalase-negative and were selected for subsequent assays (Fig. 1).

## Evaluation of functional characteristics

### Secretion of enzymes

Among the evaluated lineages (*n* = 68), 65% secreted at least one of three hydrolytic enzymes (protease, esterase, or ligninase) or exhibited the ability to solubilize phosphate (Table S2). None of the evaluated lineages secreted amylase or cellulase. Fewer lineages secreted ligninase (12%) as compared to the number of lineages secreting protease (16%) or esterase (22%) (Table S2). Approximately 34% of the lineages solubilized phosphate. The commercial probiotics used as positive controls (LL, LS, and EF) did not exhibit any of this functional trait (Table SS2).

### Antibiosis

The commercial probiotics LL and EF showed antimicrobial activity against only one of the evaluated pathogens (Table 1). Twenty-six of the evaluated lineages (38%) acted against at least one of the tested pathogens, with 26% and 21% specifically acting against *C*. *lusitaniae* and *Klebsiella* sp. 29U, respectively, while 16% were active against *P. aeruginosa* 46Ø, *Staphylococcus* sp. 100U, and *E. coli* 109U (Table 1). *L. plantarum* PA17 and PA19 stood out for their ability to inhibit all the evaluated pathogens The largest inhibition halos were observed for *L. plantarum* PA14 and PA18 against *E. coli* 109U (14.70 ± 0.60 mm and 15.30 ± 1.00 mm, respectively). The smallest inhibition halos were recorded with *Enterococcus hirae* K4 and *P. pentosaceus* PA33 against *Staphylococcus* sp. 100U (5.00 ± 1.00 mm and 5.30 ± 1.20 mm, respectively) (Table 1).

**Table 1:** Antagonistic activity against pathogenic microorganisms of potential probiotic lactic acid bacteria lineages isolated from mammals

Lineage	Origin	Pathogens				
		Klebsiella sp 29U	Pseudomonas aeruginosa 46Ø	Staphylococcus sp 100U	Escherichia coli 109U	Candida lusitaneae 163
*E. faecium* PA1	Piglet	0	0	0	0	0
*E. faecium* PA2	Piglet	0	0	0	0	0
*E. faecalis* B10	Bat	0	0	0	0	0
*E. faecalis* B16	Bat	0	0	0	0	0
*E. faecalis* C1	Calf	0	0	0	0	0
*E. faecalis* C10	Calf	0	0	0	0	0
*E. faecalis* C11	Calf	0	0	0	0	0
*E. faecalis* C12	Calf	0	0	0	0	0
*E. faecalis* C13	Calf	0	0	0	0	0
*E. faecalis* C14	Calf	0	0	0	0	0
*E. faecalis* C3	Calf	0	0	0	0	0
*E. faecalis* C9	Calf	0	0	0	0	0
*E. faecalis* K5	Cat	0	0	0	0	0
*E. faecalis* K7	Cat	0	0	0	0	0
*E. faecalis* K8	Cat	0	0	0	0	0
*E. faecalis* PA22	Piglet	0	0	0	0	0
*E. faecalis* PA24	Piglet	0	0	0	0	0
*E. faecalis* PA25	Piglet	0	0	0	0	0
*E. faecalis* PB12	Piglet	0	0	0	0	0
*E. faecalis* PB14	Piglet	0	0	0	0	0
*E. faecalis* PB27	Adult sow	10.30 ± 0.60^cde^	0	0	0	0
*E. faecalis* PB7	Piglet	0	0	0	0	0
*E. faecium* PA11	Piglet	11.00 ± 0.00^bcde^	0	0	0	9.70 ± 0.60^ef^
*E. faecium* PA13	Piglet	0	0	0	0	9.00 ± 0.00^ef^
*E. faecium* PA28	Piglet	0	0	0	8.30 ± 0.60^de^	0
*E. faecium* PA29	Piglet	0	0	0	0	0
*E. faecium* PA3	Piglet	0	0	0	0	9.70 ± 0.60^ef^
*E. faecium* PA30	Piglet	0	0	0	0	0
*E. faecium* PA35	Piglet	0	0	0	0	0
*E. faecium* PA7	Piglet	11.30 ± 0.60^bcd^	0	0	0	9.70 ± 0.60^ef^
*E. faecium* PA9	Piglet	12.00 ± 1.00^bcd^	0	0	0	9.30 ± 0.60^ef^
*E. faecium* PB9	Piglet	0	0	0	0	0
*E. hirae* B11	Bat	0	0	13.00 ± 1.70^de^	0	0
*E. hirae* C4	Calf	0	0	0	0	0
*E. hirae* C5	Calf	0	0	0	0	0
*E. hirae* K1	Cat	0	0	0	0	0
*E. hirae* K2	Cat	0	0	0	0	0
*E. hirae* K3	Cat	0	0	0	0	0
*E. hirae* K4	Cat	0	0	5.00 ± 1.00^g^	0	9.00 ± 1.00^ef^
*E. hirae* K6	Cat	0	7.30 ± 1.20^gh^	0	0	10.67 ± 0.57^def^
*E. hirae* K9	Cat	0	0	0	0	5.30 ± 1.2^h^
*L. mucosae* PB16	Piglet	0	0	0	0	0
*L. mucosae* PB17	Piglet	0	0	0	0	0
*L. mucosae* PB18	Piglet	0	0	0	0	0
*L. plantarum* B20	Bat	0	0	0	0	0
*L. plantarum* PA10	Piglet	0	0	0	0	0
*L. plantarum* PA12	Piglet	11.70 ± 0.60^bcd^	12.00 ± 0.00^d^	0	0	15.30 ± 1.20^b^
*L. plantarum* PA14	Piglet	13.30 ± 1.2^bc^	0	14.70 ± 1.20^d^	14.70 ± 0.60^bc^	12.70 ± 1.20^bcd^
*L. plantarum* PA15	Piglet	0	0	0	0	0
*L. plantarum* PA16	Piglet	0	12.30 ± 0.60^d^	8.30 ± 0.60^fg^	10.70 ± 1.20^de^	10.70 ± 0.60^def^
*L. plantarum* PA17	Piglet	11.30 ± 1.20^bcd^	8.70 ± 0.60^efg^	12.00 ± 1.00^de^	11.30 ± 1.20^cde^	10.70 ± 0.60^def^
*L. plantarum* PA18	Piglet	8.00 ± 0.00^ef^	15.30 ± 1.20^c^	15.00 ± 1.00^cd^	15.30 ± 1.20^b^	0
*L. plantarum* PA19	Piglet	9.70 ± 0.60^def^	10.30 ± 0.60^def^	10.30 ± 0.60^ef^	10.30 ± 0.60^e^	11.70 ± 1.20^cde^
*L. plantarum* PA20	Piglet	11.30 ± 0.60^bcd^	11.00 ± 1.00^de^	0	11.30 ± 0.60^de^	0
*L. plantarum* PA21	Piglet	0	0	0	0	0
*L. plantarum* PA23	Piglet	0	0	0	0	0
*L. plantarum* PA27	Piglet	12.30 ± 0.60^bcd^	0	11.00 ± 1.00^ef^	9.30 ± 0.60^e^	0
*L. plantarum* PA31	Piglet	0	0	8.30 ± 0.60^fg^	11.00 ± 1.00^de^	0
*L. plantarum* PA4	Piglet	6.70 ± 1.20^f^	10.00 ± 1.00^defg^	0	0	6.00 ± 0.00^gh^
*L. plantarum* PA5	Piglet	0	12.30 ± 0.60^d^	0	0	9.70 ± 0.60^ef^
*L. plantarum* PA6	Piglet	9.30 ± 0.60^def^	0	0	10.70 ± 0.60^e^	8.70 ± 0.6^fg^
*L. plantarum* PA8	Piglet	0	7.70 ± 0.60^fgh^	0	0	5.30 ± 1.20^h^
*L. plantarum* PB28	Adult sow	10.00 ± 0.00^de^	5.30 ± 1.20^h^	5.00 ± 1.00^g^	0	0
*P. pentosaceus* PA33	Adult sow	0	0	5.30 ± 1.20^g^	8.00 ± 1.00^e^	11.30 ± 0.60^cdef^
*S. porcorum* PA37	Adult sow	0	0	0	0	0
*S. suis* PA36	Adult sow	0	0	0	0	0
*S. suis* PB22	Adult sow	0	0	0	0	0
*W. paramesenteroides* PA26	Piglet	0	0	0	0	0
*E. faecium* EF	Commercial	0	0	0	0	12.70 ± 0.60^bcd^
*L. salivarus* LL	Commercial	0	0	0	0	1.17 ± 0.60^cde^
*L. lactis* LS	Commercial	0	0	0	0	0
Ampicillin	Antibiotic	0	0	47.30 ± 2.50^a^	0	0
Chloramphenicol	Antibiotic	44.00 ± 2.00^a^	33.00 ± 1.00^a^	18.30 ± 1.20^bc^	30.00 ± 2.00^a^	28.70 ± 0.60^a^
Tetracycline	Antibiotic	14.00 ± 2.64^b^	29.00 ± 1.70^b^	20.00 ± 0.00^b^	14.00 ± 1.70^bcd^	13.70 ± 2.30^bc^

^*Letters in the same column do not differ statistically by ANOVA followed by Tukey’s test, *p* < 0.05. Data are expressed as the mean and standard deviation of the inhibition halo diameter (mm)^

**Fig. 2:**
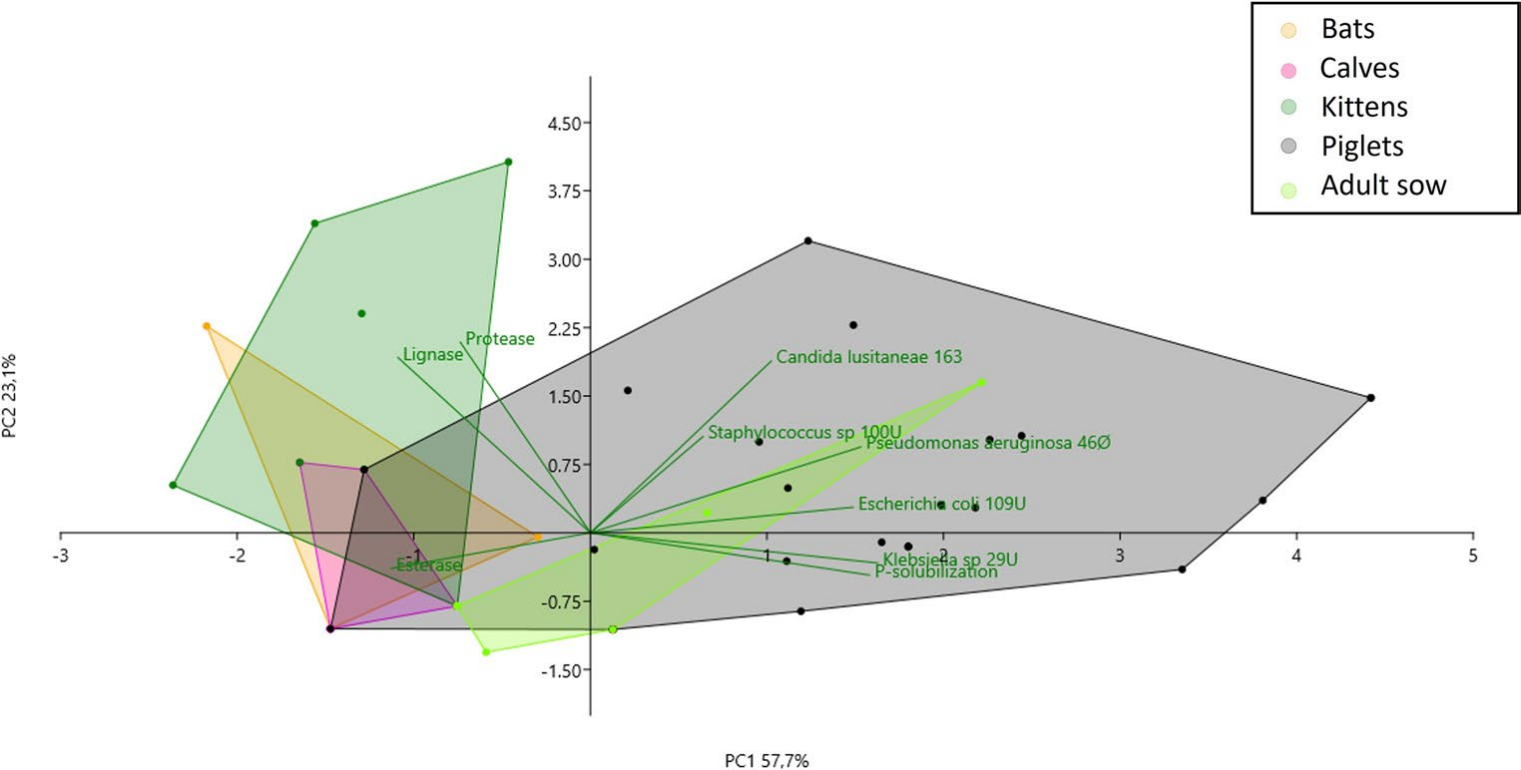
Principal Component Analysis (PCA) projection of lactic acid bacteria communities associated with mammals on the basis of functional traits

The profile of secreted enzymes, phosphate solubilization, and antibiosis varied across the LAB species and within the same LAB species, which demonstrates that lineages of the same LAB species had a distinct profile of functional traits (Table S3). In turn, LAB communities had distinct functional traits depending on the mammal they originated from (PERMANOVA, *p* < 0.0001) (Fig. 2).

Together, PC1 and PC2 axes explain approximately 79.04% of the total variation in the data, and the separation of groups indicates that functional traits of the LAB species varied across mammalian groups, especially when piglets were compared to other groups. The LAB isolated from piglets were more active against *C*. *lusitaneae* 163 and present more phosphate solubilization activity. The hierarchical clustering dendrogram shows that the LAB communities isolated from cats, bats, and calves shared greater functional similarity and differed from the LAB communities found in the adult sow and piglets (Fig. S1). Traits such as cellulase, amylase, and ligninase secretion were less pronounced, which suggests reduced functionality in the analyzed mammals (Fig. S2). We evaluated the isolated LAB lineages that presented antagonistic activity against at least one pathogen (*n* = 26) in subsequent assays.

### Cell surface properties

#### Hydrophobicity, self-aggregation, and co-aggregation with pathogens

Except for the *L. plantarum* lineages PA4, PA14, PA18, PA20, PA27, and PB28, all the other evaluated lineages and the commercial probiotics (EF, LL, and LS) exhibited low hydrophobicity levels (Table 2). The self-aggregation capacity varied across the evaluated lineages and ranged from 6.67 ± 12.58% for *E. faecalis* K7 to 90.33 ± 4.37% for *E. faecium* PA3. Co-aggregation with pathogens varied depending on the evaluated lineage and the tested microorganism species. All the evaluated lineages aggregated with the pathogens used in the assays. The co-aggregation values ranged from 16.81 ± 15.25 to 65.81 ± 5.81 for the co-aggregation of *L. plantarum* PA12 and *P. pentosaceus* PA33 with the pathogen *Staphylococcus* sp 100U and *Klebsiella* sp 29U, respectively (Table 2).

**Table 2:** Percentages of hydrophobicity, self-aggregation, and co-aggregation of different lactic acid bacteria lineages

Lineage	Hydrophobicity (%)	Self-aggregation (%)	Co-aggregation (%)				
			Klebsiella sp 29U	Pseudomonas aeruginosa 46Ø	Staphylococcus sp 100U	Escherichia coli 109U	Candida lusitaneae 163
*E. faecalis* B10	0.60 ± 0.75^f^	67.5 ± 1.80^fghi^	63.47 ± 7.43^ab^	63.99 ± 2.74^ab^	49.72 ± 8.54^ab^	50.31 ± 1.33^a^	57.5 ± 1.77^a^
*E. faecalis* K7	1.06 ± 1.25^f^	6.67 ± 12.58^n^	57.67 ± 12.04^abcd^	58.48 ± 8.99^ab^	48.80 ± 5.06^ab^	51.83 ± 1.76^a^	54.08 ± 6.8^ab^
*E. faecalis* PB27	0.48 ± 0.32^f^	78.83 ± 0.76^bcdef^	58.20 ± 7.05^abcd^	56.33 ± 7.67^abc^	45.49 ± 0.23^abcd^	47.70 ± 3.07^ab^	42.31 ± 2.35^abcd^
*E. faecium* PA11	12.79 ± 2.32^ef^	51.00 ± 7.86^jkl^	32.83 ± 6.79^de^	36.46 ± 1.21^c^	42.95 ± 11.38^abcde^	39.15 ± 1.49^bc^	51.12 ± 0.73^abc^
*E. faecium* PA13	7.96 ± 5.03^ef^	53.00 ± 4.33^ijkl^	64.22 ± 4.00^ab^	55.79 ± 1.85^abc^	27.77 ± 12.14^cdefgh^	47.65 ± 1.56^ab^	53.85 ± 2.51^ab^
*E. faecium* PA28	1.18 ± 1.25^f^	37.67 ± 4.16^lm^	57.90 ± 4.68^abcd^	59.19 ± 7.56^ab^	47.97 ± 4.94^a^	51.13 ± 2.47^a^	45.54 ± 3.3^abcd^
*E. faecium* PA3	15.73 ± 4.25^e^	90.33 ± 4.37^ab^	61.53 ± 0.54^abc^	61.87 ± 6.76^ab^	21.81 ± 4.63^fgh^	46.11 ± 3.69^ab^	55.94 ± 3.69^a^
*E. faecium* PA7	3.24 ± 4.62^ef^	67.83 ± 4.16^efghi^	53.42 ± 5.61^abcd^	48.36 ± 5.41^abc^	42.26 ± 3.20^abcdef^	49.86 ± 0.87^ab^	46.3 ± 7.79^abcd^
*E. faecium* PA9	10.42 ± 2.04^ef^	51.83 ± 5.13^ijkl^	36.14 ± 13.47^cde^	46.29 ± 3^abc^	49.67 ± 3.94^ab^	50.37 ± 3.14^a^	47.33 ± 3.47^abcd^
*E. hirae* K2	1.30 ± 0.65^f^	66.83 ± 2.93^fghij^	57.09 ± 6.25^abcd^	58.37 ± 3.51^ab^	45.49 ± 3.03^abcd^	49.13 ± 5.43^ab^	35.02 ± 10.15^cd^
*E. hirae* K4	3.62 ± 2.59^ef^	61.00 ± 6.24^ghij^	60.74 ± 8.87^abc^	54.68 ± 0.5^abc^	48.99 ± 1.77^ab^	48.58 ± 2.48^ab^	40.23 ± 9.19^bcd^
*L. plantarum* PA6	2.24 ± 2.63^f^	58.17 ± 11.47^hijk^	62.60 ± 7.01^ab^	60.61 ± 2.84^ab^	25.03 ± 3.13^defgh^	48.73 ± 2.90^ab^	53.72 ± 7.05^ab^
*L. plantarum* PA12	3.71 ± 1.01^ef^	74.17 ± 2.31^cdefgh^	39.16 ± 4.78^bcde^	44.47 ± 1.3^bc^	16.81 ± 15.25^h^	43.06 ± 5.40^abc^	32 ± 6.64^d^
*L. plantarum* PA14	67.64 ± 4.78^ab^	95.00 ± 3.28^a^	27.44 ± 10.02^e^	49.13 ± 8.18^abc^	43.01 ± 11.32^abcde^	33.41 ± 3.78^c^	40.38 ± 4.84^bcd^
*L. plantarum* PA16	2.51 ± 2.71^f^	43.83 ± 3.88^klm^	54.57 ± 10.71^abcd^	58.75 ± 3.94^ab^	47.45 ± 3.81^abc^	49.93 ± 3.82^ab^	54.23 ± 2.16^ab^
*L. plantarum* PA17	9.31 ± 2.80^ef^	81.33 ± 5.11^abcdef^	58.02 ± 3.03^abcd^	61.9 ± 6.17^ab^	41.90 ± 4.58^abcdef^	53.18 ± 11.11^a^	53.73 ± 9.97^ab^
*L. plantarum* PA18	73.39 ± 2.57^a^	83.83 ± 1.26^abcde^	56.88 ± 4.53^abcd^	63.63 ± 1.67^ab^	53.97 ± 1.70^bcdefg^	48.27 ± 1.66^ab^	49.92 ± 0.95^abc^
*L. plantarum* PA20	53.24 ± 1.35^c^	78.00 ± 1.80^bcdef^	58.05 ± 3.07^abcd^	55.42 ± 4.86^abc^	38.01 ± 1.68^bcdefg^	45.99 ± 1.52^ab^	43 ± 4.06^abcd^
*L. plantarum* PA27	59.12 ± 11.43^bc^	75.67 ± 0.76^bcdefg^	45.66 ± 19.60^abcde^	49.02 ± 23.46^abc^	53.97 ± 1.70^ab^	46.59 ± 1.74^ab^	50.15 ± 1.58^abc^
*L. plantarum* PA31	10.57 ± 3.78^ef^	79 ± 3.28^abcdef^	56.58 ± 4.21^abcd^	55.81 ± 3.54^abc^	50.88 ± 5.48^abc^	43.30 ± 0.81^abc^	50.99 ± 1.13^abc^
*L. plantarum* PA4	47.24 ± 4.09^cd^	89.50 ± 1.32^abc^	54.73 ± 5.67^abcd^	59.48 ± 3.62^ab^	19.04 ± 8.39^gh^	47.99 ± 3.92^ab^	53.26 ± 3.2^ab^
*L. plantarum* PA5	0.32 ± 0.28^f^	71.50 ± 1.32^defgh^	52.19 ± 3.37^abcde^	62.17 ± 2.2^ab^	23.40 ± 5.14^efgh^	49.33 ± 1.27^ab^	50.63 ± 4.92^abc^
*L. plantarum* PA8	3.31 ± 0.62^ef^	78.67 ± 1.89^bcdef^	63.48 ± 6.50^ab^	47.58 ± 0.6^abc^	53.37 ± 5.05^ab^	50.36 ± 0.77^a^	54.54 ± 1.97^ab^
*L. plantarum* PB28	34.70 ± 5.34^d^	69.83 ± 5.80^defgh^	62.30 ± 7.08^ab^	61.25 ± 5.78^ab^	45.94 ± 9.14^abc^	52.06 ± 3.26^a^	43.92 ± 3.43^abcd^
*L. plantarum* PA19	4.11 ± 1.68^ef^	84.67 ± 1.04^abcd^	58.66 ± 7.86^abcd^	57.32 ± 1.47^ab^	37.66 ± 1.59^bcdefg^	49.58 ± 0.47^ab^	51.05 ± 1.84^abc^
*P. pentosaceus* PA33	4.14 ± 0.64^ef^	39.20 ± 3.76^abcdef^	65.81 ± 5.81^a^	65.29 ± 6.36^a^	59.42 ± 3.45^ab^	50.49 ± 3.07^a^	56.03 ± 1.44^ab^
*E. faecium* EF	10.98 ± 11.08^ef^	7.33 ± 7.02^n^	52.01 ± 12.91^abcde^	61.12 ± 1.78^ab^	48.37 ± 4.17^ab^	44.69 ± 2.42^ab^	46.46 ± 6.73^abcd^
*L. lactis* LL *L. salivarus* LS	8.81 ± 0.07^ef^ 4.45 ± 5.98^ef^	38.17 ± 10.50^m^ 38.33 ± 7.51^lm^	50.28 ± 9.97^abcde^ 63.84 ± 3.76^ab^	64.8 ± 2.2^a^ 65.83 ± 3.4^a^	50.96 ± 4.99^abc^ 53.96 ± 7.05^ab^	49.99 ± 2.51^ab^ 52.83 ± 2.09^a^	47.68 ± 2.71^abcd^ 44.38 ± 8.38^abcd^

^*Letters in the same column do not differ statistically by ANOVA followed by Tukey’s test, *p* < 0.05^

### Tolerance to acid pH (2.5) and bile salts (0.3%)

**Fig. 3:**
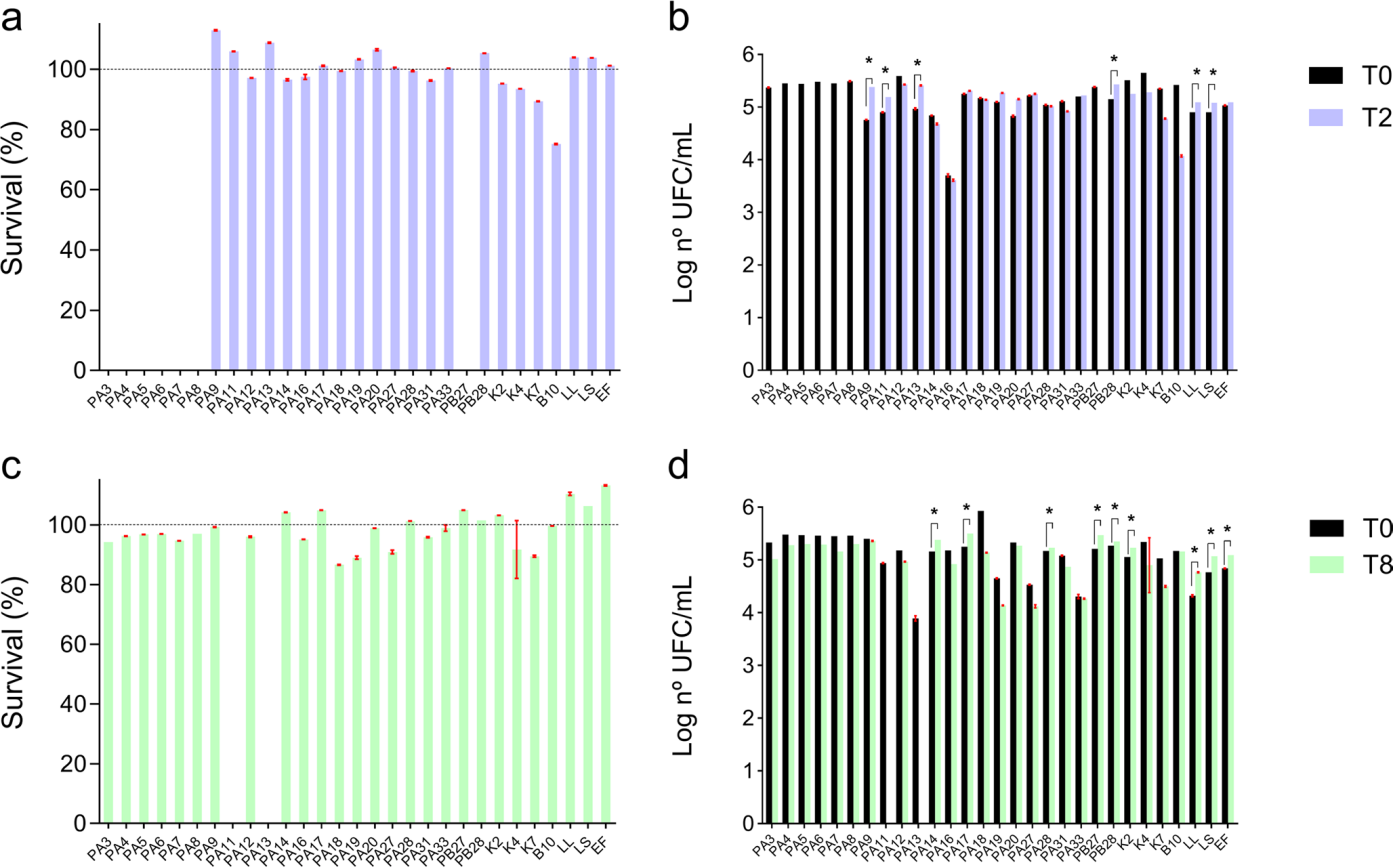
Profile of tolerance to low pH (2.5) and bile salts (0.3%) of LAB lineages. Viable CFU counts after exposure of the LAB lineages to pH 2.5 (**a**) or 0.3% bile salts (C). Survival of the LAB lineages after exposure to pH 2.5 (**b**) or 0.3% bile salts (**d**). *Indicates an increase in the number of cells at the end of incubation (paired t-test, p < 0.001)

Approximately 65.38% (17 out of 26) of the evaluated lineages survived under acidic conditions (pH 2.5) and in the presence of 0.3% bile (Fig. 3). Viable cell counts were around 4–5 log CFU/mL after exposure to pH 2.5 and 0.3% bile for 2 and 8 h, respectively (Table S4). Only seven lineages, all isolated from piglets (26.92% of the 26 evaluated lineages), did not resist exposure to pH (2.5) for 2 h (Fig. 3a). Some lineages such as *E. faecium* PA9, PA11, PA13 and *L. plantarum* PA17, PA19, PA20, PA27, and PB28 grew in acid pH, that is, the number of cells increased compared to the initial cell count (paired t-test, *p* < 0.001) (Fig. 3b). Such increase in the number of cells also occurred for the commercial probiotics LL and LS (paired t-test, *p* < 0.001). Only two lineages (*E*. *faecium* PA11 and *E. faecium* PA13) did not grow after exposure to 0.3% bile for 8 h (Fig. 3c). Additionally, *L. plantarum* PA14, PA17, and PA28; *E. faecium* PA28 and PB27; *E. hirae* K2; and the commercial probiotics EF, LL, and LS had the number of cells increased in the presence of 0.3% bile (paired t-test, *p* < 0.001; Fig. 3d).

### Susceptibility to antibiotics and hemolytic activity

The commercial probiotics EF, LL, and LS were resistant to all the evaluated antibiotics (Table S5). The susceptibility of the evaluated lineages varied: 15 lineages (58%) were resistant to all the evaluated antibiotics. The highest percentage (100%) of resistance was observed for the antibiotics ceftazidime (30 µg), gentamicin (10 µg), polymyxin b (300 IU), oxacillin (1 µg), cefoxitin (30 µg), and sulfazotim (25 µg) (Table S5). The lowest percentage of resistance (81%) was observed for the antibiotics piperacillin + tazobactam (110 µg). *L. plantarum* PA20 showed the lowest percentage of resistance (75%) to the evaluated antibiotics, followed by *L. plantarum* PA5 and PA27 and *E. faecium* PA7 and PA13 (83%) (Table S5). All the evaluated lineages were γ-hemolytic, which indicated no hemolytic activity (Fig. S3).

### Effect on the longevity of *C. elegans* BA17

**Fig. 4:**
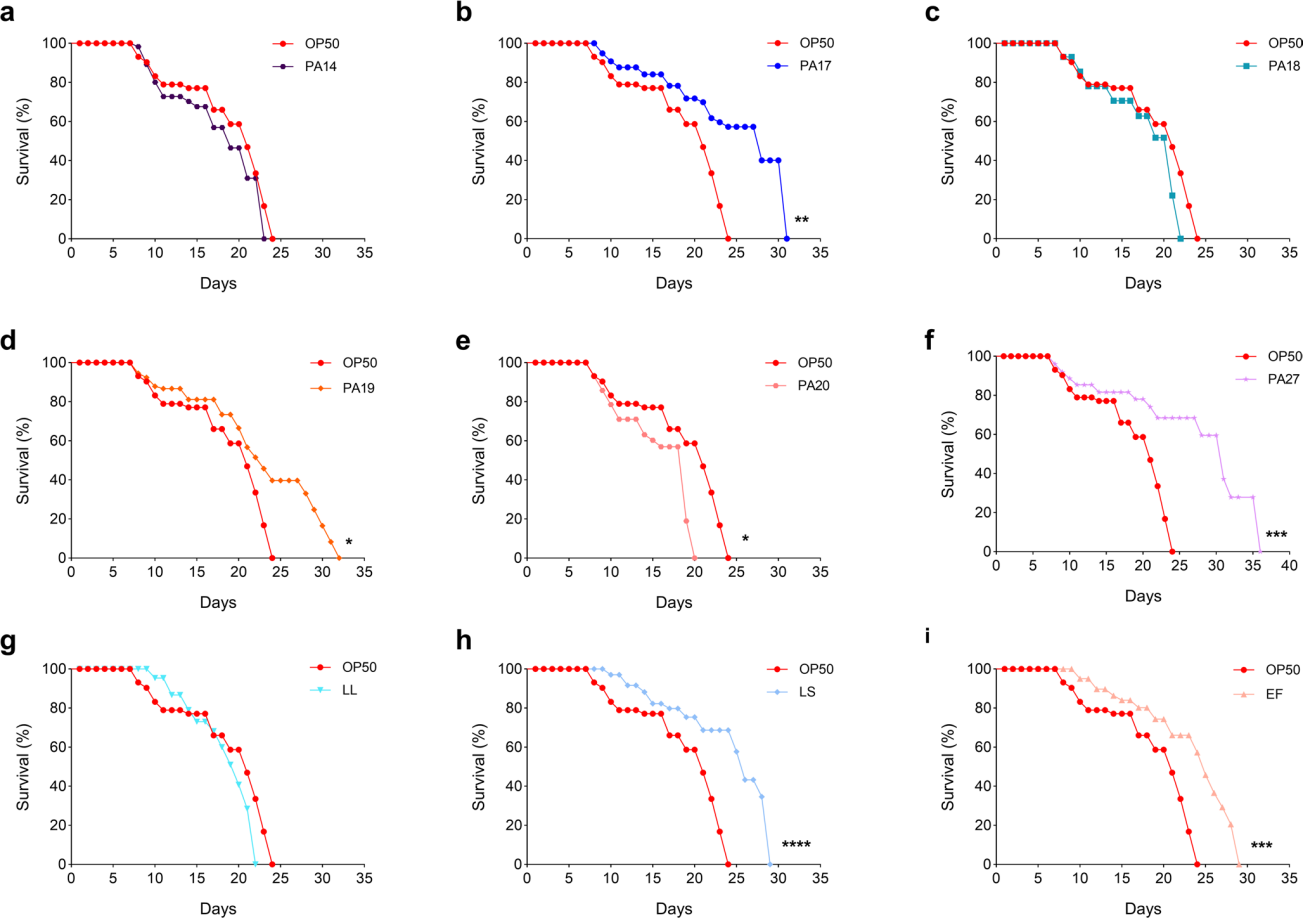
Survival curves of *C*.*elegans* BA17 fed with *L*.*plantarum* lineages, commercial probiotics (*L*.*lactis* (LL), *L*.*salivarius* (LS), or *E*.*faecium* (EF)), or *E*.*coli* OP50. Significant differences in the survival curve were determined by using the Log-rank (Mantel-Cox) test at * p < 0.05; ** p < 0.01; *** p < 0.001; and **** p < 0.0001 compared to *E*.*coli* OP5

On the basis of the in vitro assay results, we evaluated the in vivo probiotic potential of the six isolated LAB lineages that presented antimicrobial activity against four or more microorganisms, good tolerance to simulated GIT conditions, and high hydrophobicity, self-aggregation, and co-aggregation rates. Feeding *C*. *elegans* BA17 worms with different *L*. *plantarum* lineages affected survival distinctly (Table S6). The commercial probiotics EF and LS increased the lifespan of worms by 27.1% and 25.7%, respectively (Log-rank (Mantel-Cox) test, *p* < 0.05) as compared to worms fed with the negative control *E*. *coli* OP50 (Table S6). The *L*. *plantarum* PA27 lineage stood out for increasing the longevity of worms by 42.9% compared to *E*. *coli* OP50, and by 13.6%, 51.5%, and 12.4% compared to the commercial probiotics EF, LL, and LS, respectively (Log-rank (Mantel-Cox) test, *p* < 0.05; Table S6). Other lineages, such as *L*. *plantarum* PA17 and PA19, increased the lifespan of worms by 30% and 25.7%, respectively, compared to the negative control *E*. *coli* OP50 (Log-rank (Mantel-Cox) test, *p* < 0.05). In contrast, *L*. *plantarum* PA20 reduced the lifespan of worms by 15.7% compared to *E*. *coli* OP50 (Log-rank (Mantel-Cox) test, *p* < 0.05; Fig. 4).

### Effect of isolated LAB lineage on the body length and progeny of *C. elegans* N2

**Fig. 5:**
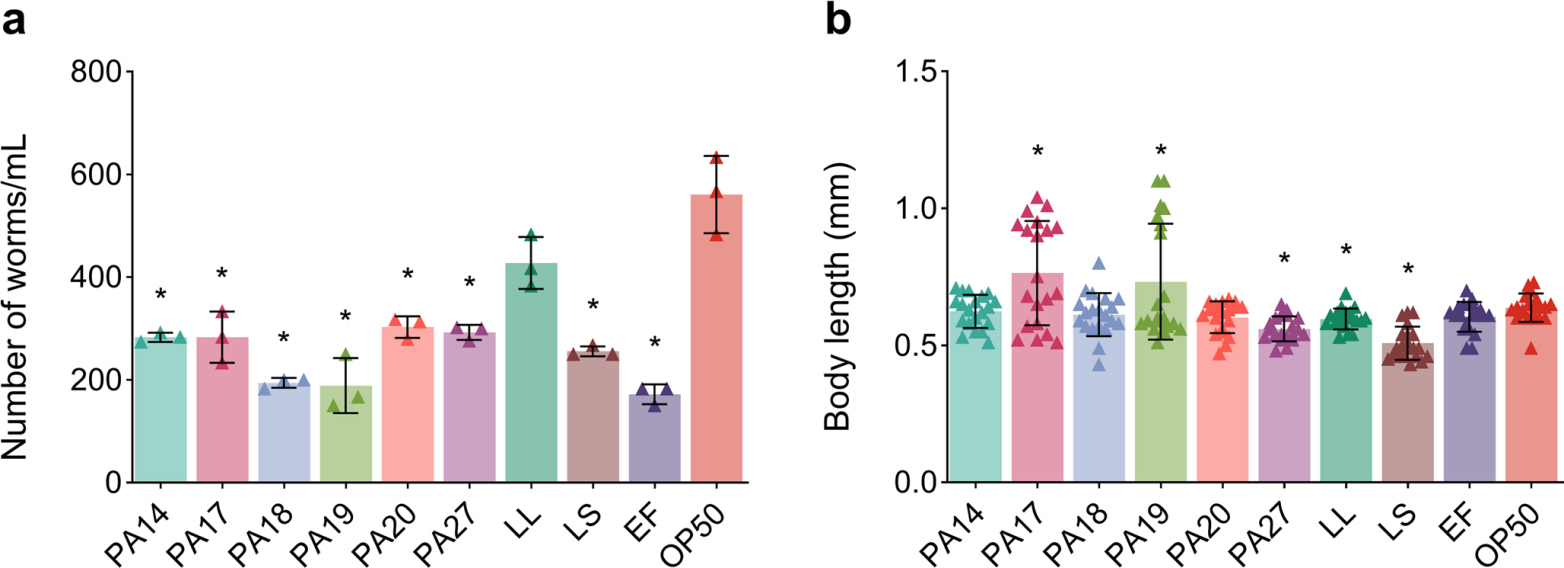
Effect of LAB isolated from mammals on the health parameters of* C.* *elegans*. (**a**) Number of adult worms and (**b**) body length of worms treated with LAB, commercial probiotics (*L*.*lactis *(LL),* L.* *salivarius* (LS), or* E.faecium *(EF)), or the negative control (*E*.*coli* OP50). * Unpaired t-test, p < 0.05 compared to the negative control* E.coli* OP50

The population densities of *C. elegans* N2 fed with different *L. plantarum* lineages were lower (158 ± 8 to 303 ± 21 worms/mL) compared to the negative control *E. coli* OP50 (561 ± 75 worms/mL) and positive controls LL, LS, and EF (428 ± 51, 256 ± 10, and 172 ± 19 worms/mL, respectively) (unpaired t-test, *p* < 0.05; Fig. 5a). Although *L. plantarum* PA19 did not increase the population density, this lineage, along with *L. plantarum* PA17, increased the body length of worms. Worms fed with these two lineages showed longer body length (0.73 ± 0.21 mm and 0.76 ± 0.19 mm, respectively) than worms fed with *E. coli* OP50 (0.66 ± 0.05) or a commercial probiotic (LL, LS, or EF, 0.60 ± 0.04 mm, 0.51 ± 0.06 mm, and 0.64 ± 0.05 mm, respectively) (unpaired t-test, *p* < 0.05; Fig. 5b).

### Effect of LAB on genes related to longevity of *C. elegans*

**Fig. 6:**
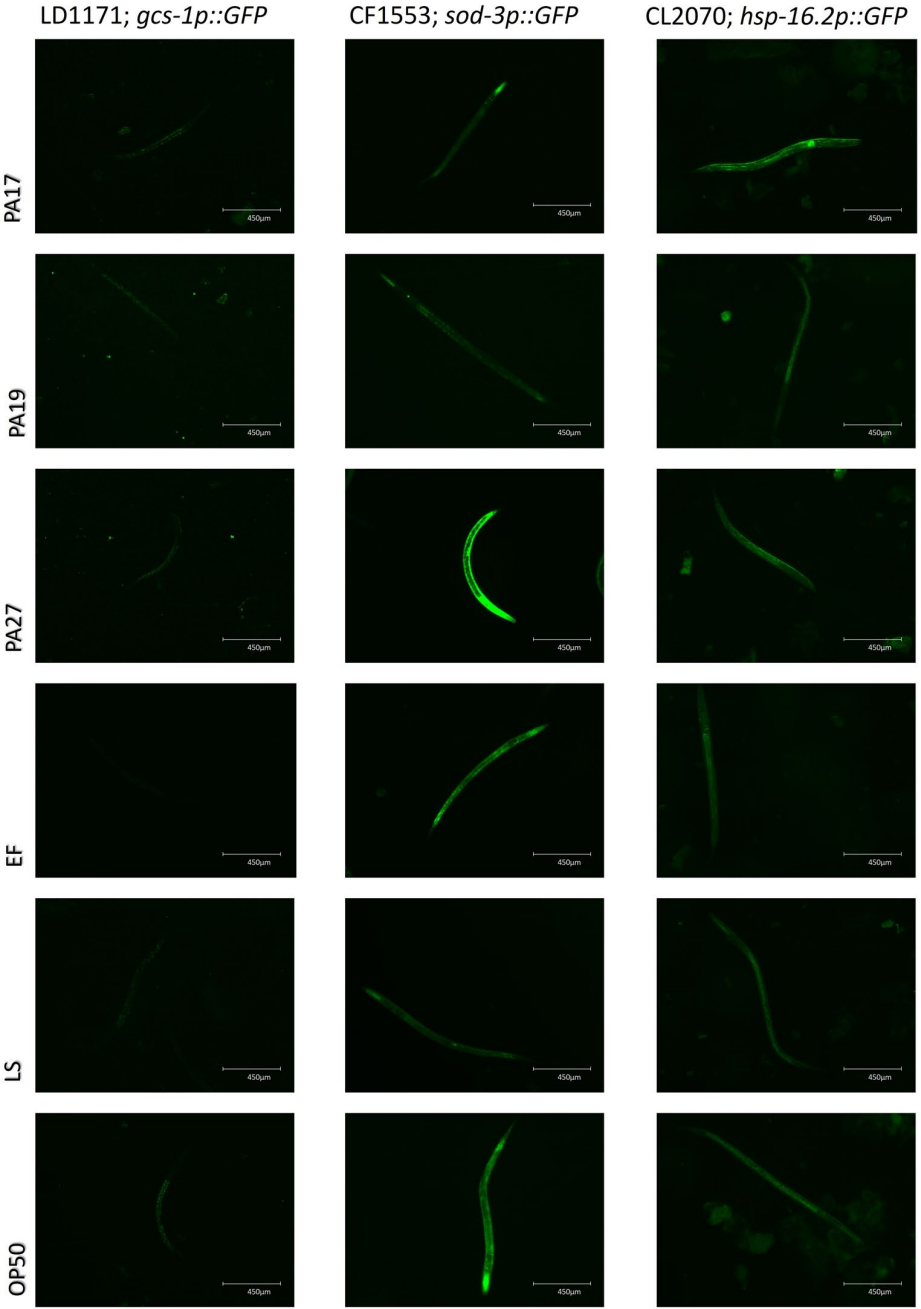
Schematic representation of worms treated with LAB strains or controls, showing the expression of enzymes related to oxidative and thermal stress in*C*.*elegans* LD1171, CF1553 and CL2070

**Fig. 7:**
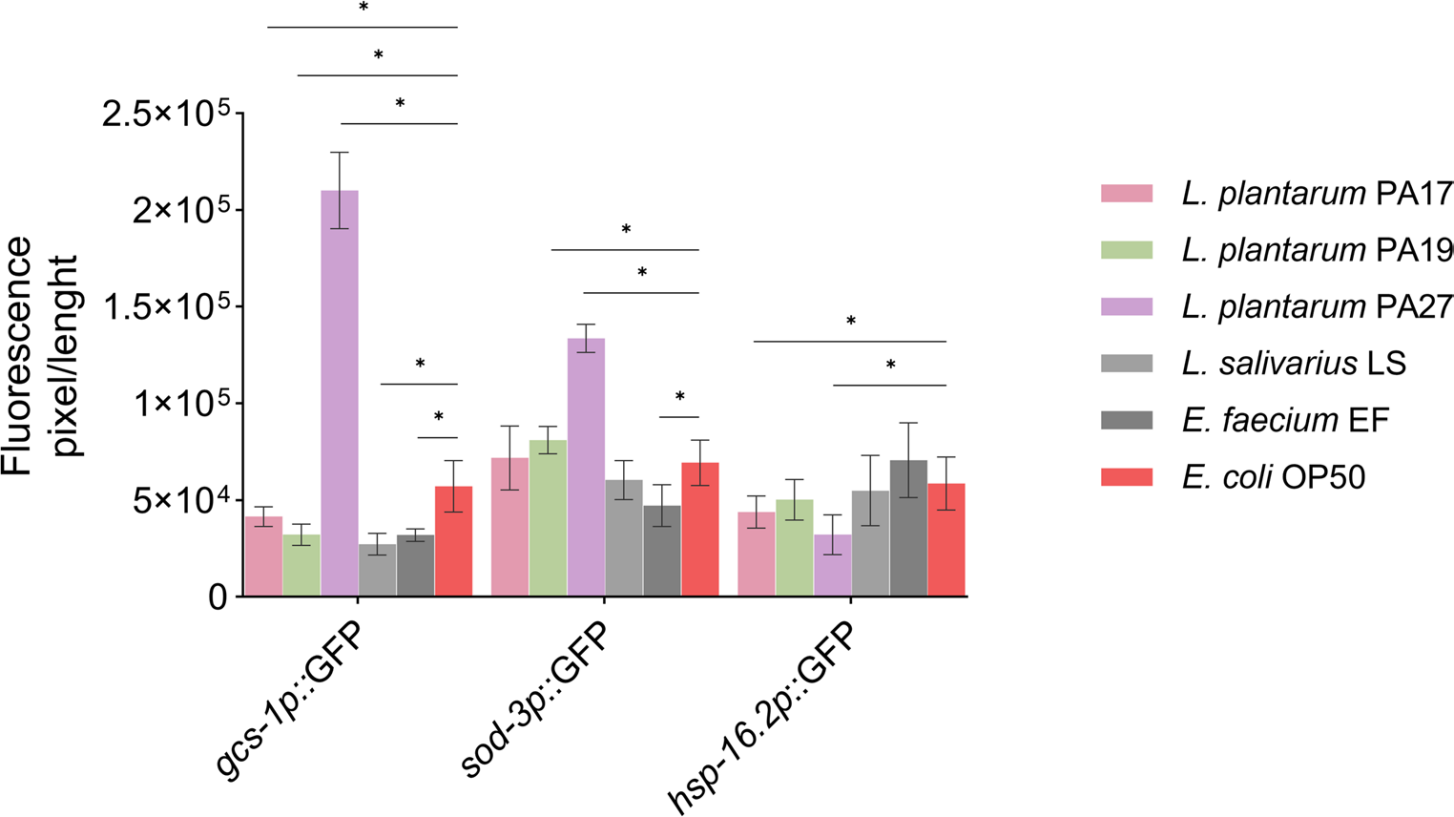
Effect of LAB lineages on the induction of genes related to oxidative and thermal stress in* C.* *elegans*.* Unpaired t-test, p < 0.05, compared to the negative control* E.* *coli* OP50

We evaluated how the lineages that increased the lifespan of worms affected the expression of genes related to oxidative stress (*gcs-1p* and *sod-3p*) and thermal stress (*hsp-16.2p*) in *C. elegans* (Fig. 6). Treatment with *L. plantarum* PA27 increased the *gcs-1p* activity: the fluorescence of the worms treated with this lineage increased by 367 ± 34% compared to worms fed with the negative control *E. coli* OP50 (unpaired t-test, *p* < 0.05; Table S7). Worms fed with *L. plantarum* PA19 or PA27 had increased *sod-3p* expression—fluorescence increased by 104 ± 24% and 192 ± 10%, respectively, compared to worms treated with *E. coli* OP50 (unpaired t-test, *p* < 0.05; Table S7). None of the evaluated lineages increased *hsp-16.2p* expression when compared to *E. coli* OP50 (unpaired t-test, *p* < 0.05; Table S7). Feeding with *L. plantarum* PA17 resulted in a distinct gene expression pattern in *C. elegans*, that is, *gcs-1p* and *sod-3p* expression increased, while *hsp-16.2p* expression had the greatest reduction among the evaluated LAB lineages (Fig. 7).

### Influence of probiotics on the activity of the DAF-16 transcription factor

**Fig. 8:**
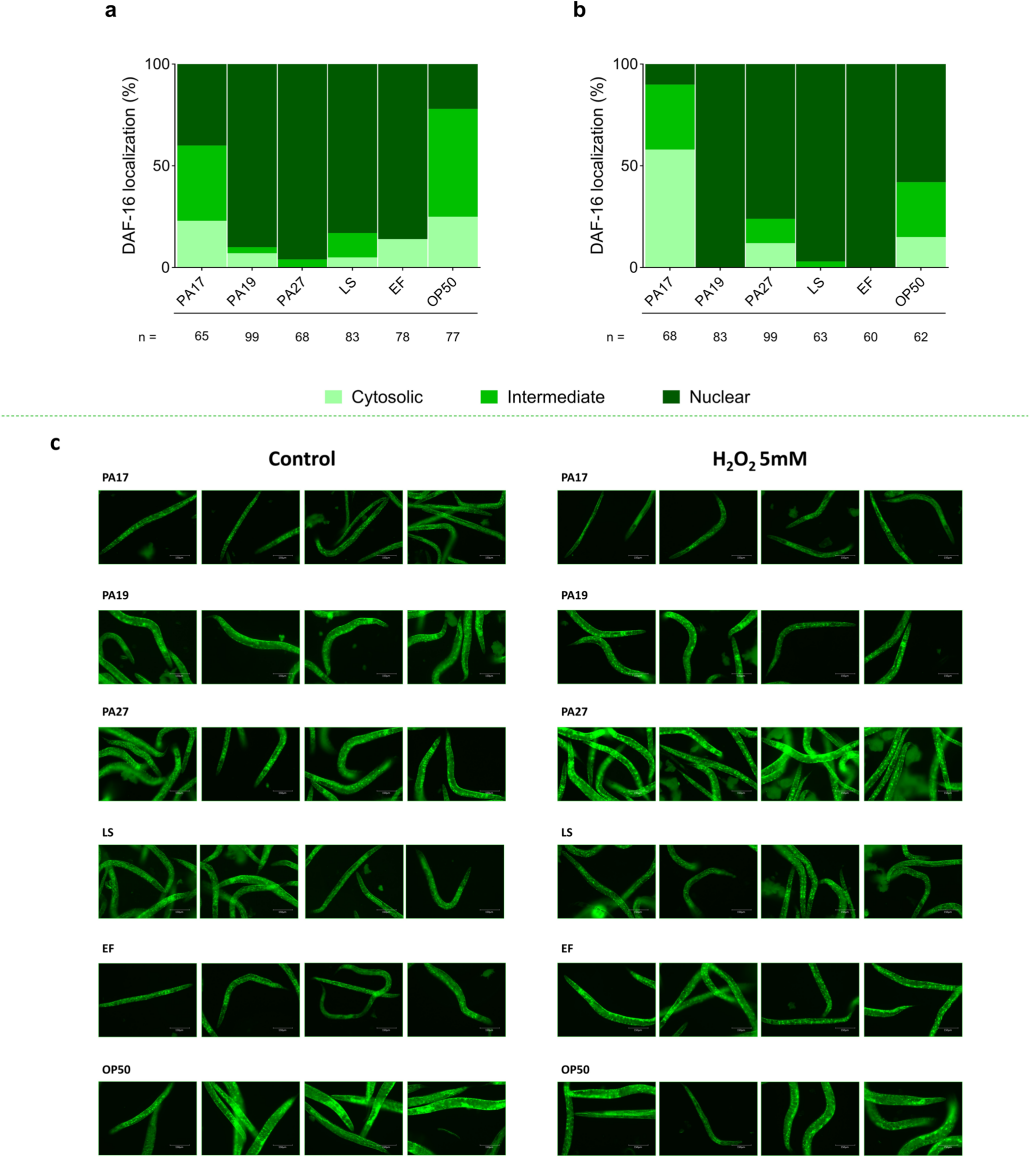
Cellular localization of DAF-16 in the *C*.*elegans* TJ356 lineage after treatment with lactic acid bacteria lineages with probiotic potential. Percentage of DAF-16 localization in each cellular location: cytosolic, intermediate, or nuclear (**a**) without oxidative stress or (**b**) under oxidative stress - 5 mM H_2_O_2_ and (**c**) image scheme of a worms treated with LAB lineages or with controls

Compared to worms fed with *E. coli* OP50, the nuclear fraction of DAF-16 increased in worms fed with *L. plantarum* PA17, PA19, or PA27 (unpaired t-test, *p* < 0.05), i.e., from 23 ± 7% to 40 ± 2%, 92 ± 4%, and 96 ± 0%, respectively, (Fig. 8a and Table S8). Under oxidative stress induced by H_2_O_2_ (5 mM), the nuclear fraction of DAF-16 increased upon treatment with *L. plantarum* PA19 or PA24 (unpaired t-test, *p* < 0.05): from 59 ± 51% in the negative control treatment, *E. coli* OP50, to 100% and 75.8%, respectively (Fig. 8b and Table S8).

## Discussion

In this study, we have characterized LAB isolated from fecal samples of mammals (piglets, calves, cats, or bats) or from the vaginal mucosa or teat skin samples of a lactating adult sow. Some of the isolated LAB exhibited important in vitro probiotic functional traits and increased longevity in the *C. elegans* animal model. Our data corroborate the data of other studies that have demonstrated that mammalian LAB positively affect animal health (Zhong et al. 2022).

The diversity of LAB present in the feces of the healthy mammals (piglets, calves, cats, and bats) and in the vaginal mucosa and teat skin of the lactating adult sow reflects the frequently observed heterogeneity of LAB communities obtained from different mammalian hosts (George et al. 2018). Our data indicates that these animals were associated with bacteria belonging to the phyla Bacillota, Pseudomonadota, or Actinomycetota as revealed by culture-dependent methods. The phyla Bacillota and Actinomycetota have been described in the intestinal microbiota of piglets, specifically in samples obtained from the ileum, colon, and feces (Dong et al. 2022), and in fecal samples obtained from conventionally raised cats (Minamoto et al. 2012). In the samples obtained from *Artibeus lituratus* (family Phyllostomidae), the phyla Pseudomonadota and Bacillota were the most abundant, which agrees with the findings of Carrillo-Araujo et al. (2015) for other bats of the same family. In samples obtained from calves, the phyla Bacillota and Pseudomonadota predominate in the gastrointestinal tract (GIT) (Kim et al. 2024b), as also verified in the present study.

Analyses of the diversity of the 11 bacterial genera found in our samples reinforce the variability among the studied hosts and highlight the frequently observed heterogeneous diversity of LAB in different mammalian hosts (Huang et al. 2020). In the fecal samples obtained from bats (*A. lituratus*), *Escherichia* was the most abundant genus (50%), followed by *Enterococcus* (30%) and *Klebsiella* (10%), genera that are widely associated with the microbiota of frugivorous and insectivorous animals (André et al. 2023). Furthermore, the presence of the genera *Escherichia*, *Enterococcus*, and *Klebsiella* in the microbiota of *A. lituratus* is consistent with findings in other mammals (Silva et al. 2012). Although these genera are associated with proper functioning of the digestive system and are therefore frequently detected in different animal groups, including mammals (Dubin and Pamer 2018), some species and lineages can act as opportunistic pathogens in animals and humans (Nowakiewicz et al. 2020, 2021; Bazzoni et al. 2024).

In calves (*Bos taurus taurus*), the genus *Enterococcus* predominated (71%), which is consistent with studies associating this genus with immunological modulation and digestion in the GIT of young ruminants (Gomez et al. 2019). Under a dysbiotic condition, as in the case of diarrheic calves, the microbiota is enriched with *Enterococcus*, which evidences the adaptive role of this genus in altered intestinal environments. This ability to thrive in adverse scenarios highlights that this genus is important for interaction with the host’s gastrointestinal environment and modulation of immune responses (Gomez et al. 2022).

The genus *Enterococcus* plays a significant role in the initial colonization of the GIT of newborn cats. *Enterococcus*, especially *Enterococcus hirae*, is common in the intestinal microbiota of healthy young cats, contributes to the formation of a balanced intestinal microbiota, and aids in digestion and immune system modulation (Ghosh et al. 2013). Moreover, the genus *Enterococcus* is commonly found in other mammalian groups (Minamoto et al. 2012). These pieces of evidence corroborate the observation that *Enterococcus* was exclusive (100%) to newborn cats (*Felis catus*) and stood out for its initial prevalence in intestinal colonization.

In piglets aged 5 to 40 days (*Sus scrofa domesticus*), *Enterococcus* (36%) and *Lactiplantibacillus* (34%) were present at high proportions, which indicates a robust and functional microbiota. In turn, *Staphylococcus* (14%) appeared in lower abundance. *Lactobacillus* (related to *Lactiplantibacillus*) and *Enterococcus* are dominant genera in the intestinal microbiota of pigs analyzed by culture-dependent methods(Wang et al. 2021). This pattern suggests that these genera play essential roles in digestion and intestinal microbiota modulation during early developmental stages. The vaginal mucosa and teat skin of the lactating adult sow (*Sus scrofa domesticus*) were predominantly colonized by the genera *Staphylococcus* (40%) and *Streptococcus* (20%), which are frequently detected in pig epidermis (Kemper and Preissler 2011; Strube et al. 2018) and other mammals (Otto 2010; Misic et al. 2015; Ogura et al. 2022). These differences in relative abundance reflect microbial adaptations to the hosts’ diet, age, and environment and reinforce the specific functional role of each genus.

Analysis of the relative abundance of species revealed specific colonization patterns across the mammalian groups. *E. coli* (50%), *E. faecalis* (20%), *E. hirae* (10%), and *Klebsiella pneumoniae* (10%) reflect a functional composition that is possibly associated with digestive adaptations related to the flying lifestyle of the bat *A. lituratus*. Bats possess distinct intestinal microbiotas that are often enriched with bacteria of the phylum Pseudomonadota and a digestive physiology that can restrict the composition of the microbiome. These characteristics can be understood as adaptations for optimizing the extraction of nutrients from specific diets, as suggested by Nishida and Ochman (2018). Nevertheless, *E. coli* and species of the genus *Enterococcus* are known for their ability to act as opportunistic pathogens and are associated with human and animal diseases in specific cases (Nowakiewicz et al. 2020, 2021). The presence of *E. coli* in high abundance in frugivorous bats, as observed in this study, may reflect its ability to adapt to the intestinal environment of these mammals, but potential risks associated with multidrug-resistant or pathogenic lineages must be investigated.

The high abundance of *E. faecalis* and *E. hirae* in calves (*Bos taurus taurus*) and newborn cats (*Felis catus*) indicates that *Enterococcus* species play a role in digestive processes and in the initial formation of the intestinal microbiota and neonatal immunological adaptation. *E. faecalis* modulates inflammatory responses by suppressing secretion of pro-inflammatory cytokines, such as IL-8, through the JNK and p38 signaling pathways (Wang et al. 2014). Additionally, *E. faecium* and *E. faecalis* are predominant intestinal colonizers in neonatal mammals and contribute to stabilizing the microbiota in the first days of life (Hufnagel et al. 2007). Besides *E. faecium* and *E. faecalis*, *L. plantarum* was an abundant species in the anal mucosa of piglets (*Sus scrofa domesticus*), while *Staphylococcus haemolyticus*, *Staphylococcus simulans*, and *Kurthia populi* stood out in the microbiota of the vaginal mucosa and teat skin of the lactating adult sow. This composition suggests that the microbiota adapted to different ecological niches (Yu et al. 2024; Chen et al. 2025).

The isolated LAB lineages exhibited diversified functional traits, such as the ability to secrete hydrolytic enzymes (ligninase, protease, or esterase) and to solubilize phosphate. These characteristics are associated with modulation of the intestinal microbiota and digestion of food (Zhang et al. 2015). Most (65%) of the isolated lineages exhibited at least one of these traits, regardless of the mammal they originated from (Ruiz Rodríguez et al. 2019). *L. plantarum* (PA3, PA4, PA5, and PA16), isolated from piglets, stood out for secreting esterase and solubilizing phosphate, which are considered important traits for maintaining intestinal homeostasis (Bhatia et al. 2022). The enzymes secreted by these bacteria play a significant role in the biotransformation of phenolic compounds, such as hydroxybenzoic and hydroxycinnamic acids, so that the released bioactive derivatives are more easily absorbed and used by the organism (Landete et al. 2021). The fact that the lineages did not secrete amylase or cellulase contrasts with studies in environments such as fermented foods and soil and highlights the structural and functional differences between bacterial communities in response to the hosts’ characteristics (Kieliszek et al. 2021).

The inclusion of young animals from different species was justified because all were within the early-life developmental phase, during which the gut microbiota undergoes rapid and comparable maturation across mammals (Laforest-Lapointe and Arrieta 2017; Jain 2020; Choudhury et al. 2021; Quan et al. 2023). Although ages varied, factors such as colostrum intake, suckling, and early environmental exposure similarly influence microbial establishment in piglets, calves, and kittens. The adult lactating sow was included as a reference for a mature microbiota and as a maternal source of microorganisms relevant to the piglets sampled (Lutz et al. 2019). In bats, only adult animals were available for collection. Altogether, this sampling strategy strengthened the ecological interpretation of microbiota patterns by encompassing hosts in comparable developmental contexts or with relevant maternal roles.

Bacteria belonging to the family Lactobacillaceae have been considered safe (Qualified Presumption of Safety - QPS or Generally Recognized As Safe - GRAS) for human consumption, with several species being probiotic for humans (Koutsoumanis et al. 2023). The LAB we obtained from the animals did not show hemolytic activity, which indicates low pathogenic potential and corroborates evidence of their safety.

Probiotic bacteria can antagonize pathogenic bacteria by reducing the luminal pH, to inhibit bacterial adhesion and translocation, or producing antibacterial and defensive substances such as bacteriocins (Harzallah and Belhadj 2013). Our data indicates that some LAB we isolated from mammalian feces effectively inhibited multidrug-resistant pathogens, which highlight their potential application as probiotics to control intestinal infections. This finding is significant if we consider that antibiosis is a crucial trait when selecting new probiotics, and that the ability of a probiotic to inhibit pathogens is vital for promoting host’s health and maintaining microbial balance in the GIT (Kaur et al. 2021). The antibiosis activity of probiotics helps to prevent infections, to improve overall intestinal health, and to strengthen the host’s immune response (Prajapati et al. 2024).

*L. rhamnosus* CRL2244 exhibits antagonistic activity and antibiotic synergy against multidrug-resistant pathogens, including *Acinetobacter baumannii* (Rodriguez et al. 2024). In addition, characterization of bacteriocin-like inhibitory substances in LAB lineages obtained from fermented foods has demonstrated activity against multiple pathogens (Thuy et al. 2024). Approximately 38% of the isolated LAB lineages inhibited at least one of the tested pathogens, with *L. plantarum* (PA17 and PA19 – isolated from 5-day-old piglet feces) showing a broad range of inhibition (*Klebsiella* sp 29U, *P*. *aeruginosa* 46Ø, *Staphylococcus* sp 100U, *E*. *coli* 109U, and *C*. *lusitaneae* 163), whereas the commercial controls LL, LS, and EF inhibited only one pathogen (*C*. *lusitaneae* 163). The antibiosis activity of probiotics helps to prevent infections, to improve overall intestinal health, and to strengthen the host’s immune response (Prajapati et al. 2024). In the future, the antibiosis capacity must be evaluated in animal models to confirm this functional trait in vivo.

Most (65%) of the LAB evaluated in this study survived under acid pH (2.5) and bile salt (0.3%) conditions, which is crucial for probiotics (Wang et al. 2018). Ruiz et al. (2013) pointed out that LAB have mechanisms to resist adverse environments, such as acid pH and bile salts. These characteristics are essential for probiotics to survive in the acidic and high bile salt environment of the GIT.

Most LAB we obtained from the analyzed mammals possessed important cell surface characteristics. These characteristics are necessary for the probiotic capacity of a microorganism because they are directly related to how effectively the probiotic adheres to intestinal epithelial cells (Reuben et al. 2019). Hydrophobicity is a crucial factor for probiotics to adhere to and to colonize the GIT (Wu et al. 2023). Some studies have highlighted that probiotic self-aggregation and co-aggregation are important for protective biofilms to form (Amenu and Bacha 2023). These biofilms are essential for probiotics to resist adverse conditions in the GIT (das Neves Selis et al. 2021).

LAB synthesizes a series of metabolites that can keep the balance and homeostasis of the intestinal microbiota, to maintain the intestinal epithelial barrier, resistance to pathogens, and immune system regulation. In turn, the host’s nutrition, health, and behavior are impacted (Tang et al. 2023). Evidence suggests that host-specific LAB exist, e.g., bacteria can more efficiently colonize the host tissue from which they were isolated (Yuki et al. 2000). Moreover, LAB are more resistant to specific conditions of the GIT from which they were obtained (Saarela et al. 2000). Furthermore, some LAB are specific for folate synthesis (Levit et al. 2021). The analyzed functional trait profile separated host-dependent LAB groups. These groupings may reflect the adaptability of LAB to the different characteristics of each host’s GIT, such as pH and the presence of bile salts, among others.

Studies have shown that LAB promote health and longevity in animal models, demonstrating that they are potential probiotic agents (Matsumoto and Kurihara 2011; Zhao et al. 2013; Kim et al. 2024a; Kumar et al. 2024; Wu et al. 2025). In general, the longevity in animal models promoted by LAB is associated with mechanisms that modulate antioxidant pathways, reduce oxidative stress, or promote neuroprotection (Kumar et al. 2022; Kumaree et al. 2023; Zou et al. 2024). Such evidence reinforces the role played by LAB not only in maintaining intestinal health, but also in acting as systemic modulators that can positively impact the host’s longevity and overall well-being. This expands their potential application as functional probiotics in different biological contexts. Feeding *C. elegans* with the selected LAB lineages increased the longevity of the nematodes compared to worms fed with the standard food *E. coli* OP50, thereby indicating the potential of these lineages to promote health and to prolong life. To elucidate the mechanisms associated with this effect, we investigated the expression of genes involved in response to oxidative stress, a central pathway in aging regulation.

Our fluorescence data revealed that *L. plantarum* PA17, PA19, and PA27 promoted longevity in *C. elegans*, possibly by modulating the expression of the *gcs*−1 and *sod*−3 genes, whose regulation is associated with the transcription factors SKN-1 and DAF-16, respectively. Fluorescence data from genes labeled with GFP have been used in the literature to evaluate possible mechanisms of action of probiotics (Schifano et al. 2019; Zhang et al. 2022; Balaguer et al. 2023; Stover et al. 2023). In the future, analyses with quantitative PCR or Western blot may corroborate this data. DAF-16, which is homologous to transcription factors belonging to the forkhead O (FoxO) class in mammals, is a well-established regulator of longevity in *C. elegans* and acts on antioxidant and cell maintenance genes (Lin et al. 1997; Hsu et al. 1999; Zečić and Braeckman 2020; Balaguer et al. 2023). DAF-16 activation was corroborated by its translocation to the nucleus in treated *C. elegans* TJ356, which suggests that *L. plantarum* PA17, PA19, and PA27 promoted longevity by activating antioxidant and cell defense pathways. Researchers have obtained similar results in *C. elegans* fed with *L. plantarum* JBC5, isolated from fermented food. The action of *L. plantarum* JBC5 on longevity has been associated with its modulation of the antioxidant, innate immunity, and serotonin signaling pathways (Kumar et al. 2022). Similarly, *L. plantarum* A72 has been reported to delay aging and to prolong the lifespan of *C. elegans* by interfering with the transition of genes related to oxidative stress, such as *sod-5* and *hsp-16.1*, and inhibiting genes associated with fatty acid synthesis, such as *fat-6* and *lips-17* (Zou et al. 2024). Likewise, at an OD600_nm_ of 0.5, *Lactobacillus paracasei* HII01 originating from Chiang Mai University, Thailand, has been described to increase the lifespan and to promote neuroprotection in *C. elegans* (Kumaree et al. 2023). Although caloric restriction can extend lifespan in *C. elegans* through DAF-16 activation (Huayta et al. 2023) the longevity observed in our study is attributable to the probiotic effects of LAB. Our experimental conditions included excess food, and both *E. coli* OP50 and LAB were provided at consistent concentrations, ruling out caloric limitation as a confounding factor.

Analysis of the expression of genes related to antioxidant activity in *C. elegans* indicates that exposure to *L. plantarum* PA27 promoted *gcs-1* and *sod-3* expression. This suggests that *L. plantarum* PA27 was able to activate cellular protection mechanisms against oxidative stress. This is consistent with previous studies associating activation of these pathways with increased longevity and tolerance to stress in *C. elegans* (Roselli et al. 2019; Kaur et al. 2021).

Moreover, we observed that expression of *hsp-16.2*, a gene regulated by HSF-1 and associated with response to thermal and proteotoxic stress, decreased. This may reflect a preventive action of LAB lineages: by maintaining protein homeostasis, they prevent compensatory responses to the accumulation of damaged proteins from being activated, as described in contexts of cell protection induced by probiotics (Bron and Kleerebezem 2011). Increased antioxidant activation without heat response being induced suggests that LAB provide basal protection against environmental stress, helping homeostasis to be maintained.

*C. elegans* fed with the evaluated LAB lineages accumulated more DAF-16 in the nucleus, which corroborated our results regarding the expression of genes related to oxidative and thermal stress. In the absence of stress events, DAF-16 remains in the cytoplasm. Upon activation, it is translocated to the cell nucleus (Thiruppathi et al. 2024). Desaka et al. (2021)investigated how *Streptococcus thermophilus* (isolated from fermented dairy products) affects *C. elegans* longevity, to find that feeding with this bacterium increased the expression of *daf-16* and downstream antioxidant genes. Besides that, pro-longevity effects were reduced in *daf-16* loss-of-function mutants, which suggests that *S. thermophilus* extends the lifespan of *C. elegans* by activating the DAF-16-mediated antioxidant pathway. Another study has demonstrated that *Bifidobacterium longum* BB68 increases the longevity of *C. elegans* by regulating DAF-16-mediated innate immune signaling. This same study showed that feeding with BB68 induces nuclear localization of DAF-16 and increases the expression of the target gene *sod-3*, which indicates that the physiological effects of BB68 in *C. elegans* are mediated by DAF-16 activation (Zhao et al. 2017). These findings indicate that our isolates activated DAF-16 even under basal conditions, suggesting enhanced cellular preparedness and resistance to stress, regardless of the presence of an external stressor. In the future, transcriptome data, quantitative PCR, or RNAi will corroborate our results obtained by fluorescence analysis of genes of interest labeled with GFP.

## Conclusion

This study highlights the probiotic properties of the isolated LAB lineages associated with mammals evaluated in the *C. elegans* animal model. Furthermore, the conducted assays confirm that the host species influences LAB communities and functionalities. Among the evaluated LAB lineages, *L. plantarum* PA27 (isolated from piglets) has proven to promote longevity and to improve health-related parameters in *C. elegans* the most effectively as compared to the standard food *E. coli* OP50 and commercial probiotics (*L. lactis*, *L. salivarius*, and *E. faecium*). Although the beneficial effects observed in worms may not suffice to guarantee probiotic function in humans, the positive results are an important prerequisite for the rational selection of probiotic lineages. This work opens opportunities for future investigations into how probiotics can help to promote health through their anti-aging action and the mechanisms associated with this action. Since aging is a complex phenomenon modulated by various factors, our findings corroborate the literature indicating that the appropriate type of diet could positively remodel human health.

## Supplementary Information

Below is the link to the electronic supplementary material.

Supplementary Material 1 (DOCX 265 KB)

Supplementary Material 2 (XLSX 42.4 KB)

Supplementary Material 3 (JPG 190 KB)

Supplementary Material 4 (JPG 186 KB)

Supplementary Material 5 (JPG 490 KB)

## Data Availability

Further data that support the findings of this study are available on request from the corresponding author. Sequences are available at GenBank, as described in the Material and Methods section.
