# Supplementary material for: Lactic acid bacteria isolated from mammalian feces exhibit distinct diversity and probiotic traits
Source: World J Microbiol Biotechnol. 2026 Mar 10;42(3):128. doi: 10.1007/s11274-026-04801-8 (PMC12971750; doi:10.1007/s11274-026-04801-8)

Supplementary information

Lactic acid bacteria isolated from mammalian feces exhibit distinct diversity and probiotic traits

Maria Isabela da Silva Figueiredo^1^; Ivani Souza Mello^1^; Luana de Guimarães Bueno^1^; Risya Regina Westphal Mendes^1^; Jonathan Mádson dos Santos Almeida^2^; Alan Eriksson^3^; Gilvan Ferreira da Silva^4^; Marcos Antônio Soares^1*^

^1^ Laboratory of Biotechnology and Microbial Ecology, Institute of Biosciences, Federal University of Mato Grosso, Cuiabá, MT, Brazil

^2^ Department of Animal Science and Rural Extension, Faculty of Agronomy and Animal Science, Federal University of Mato Grosso, Cuiabá, MT, Brazil

^3^ Department of Biology and Zoology, Institute of Biosciences, Federal University of Mato Grosso, Cuiabá, MT, Brazil

^4^ Laboratory of Molecular Biology, Embrapa Western Amazon, Manaus, AM, Brazil

*Correspondent author

drmasoares@gmail.com

Fig. S1. Similarity dendrograms of bacterial species composition in bacterial communities isolated from mammals. All bacterial species (a) and Lactic acid bacteria (LAB) species (b). Cluster analysis (UPGMA) based on the Jaccard coefficient

Fig. S2. Heatmap and hierarchical clustering of functional traits and microbial communities across mammalian groups, based on Euclidean distance

Fig. S3. Hemolytic activity of BAL isolates


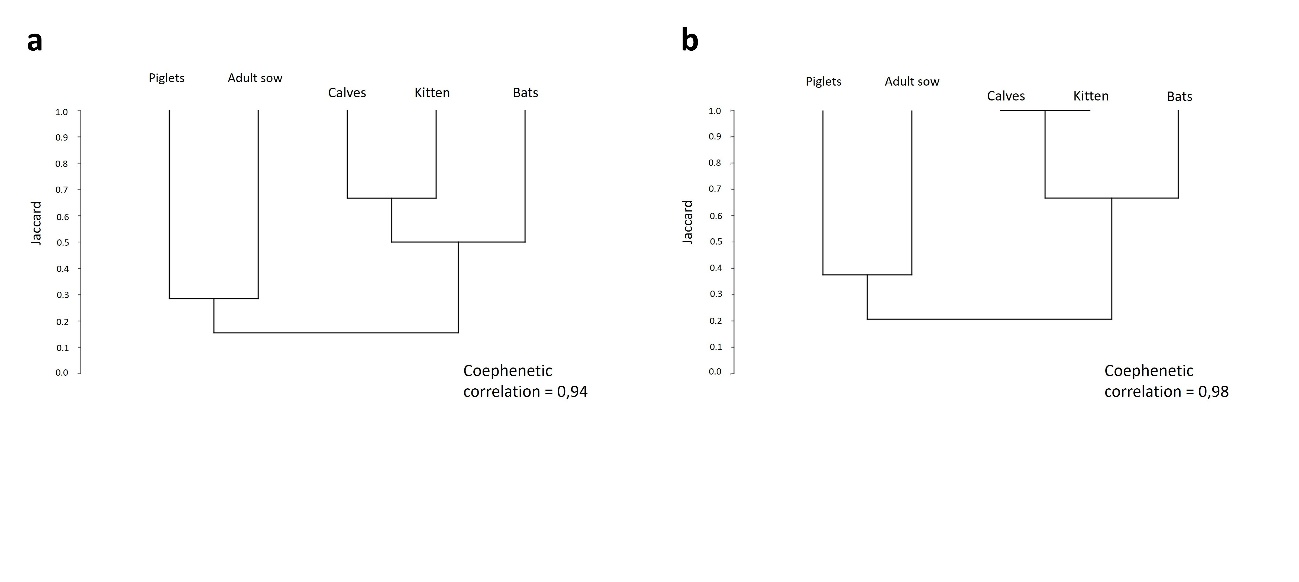


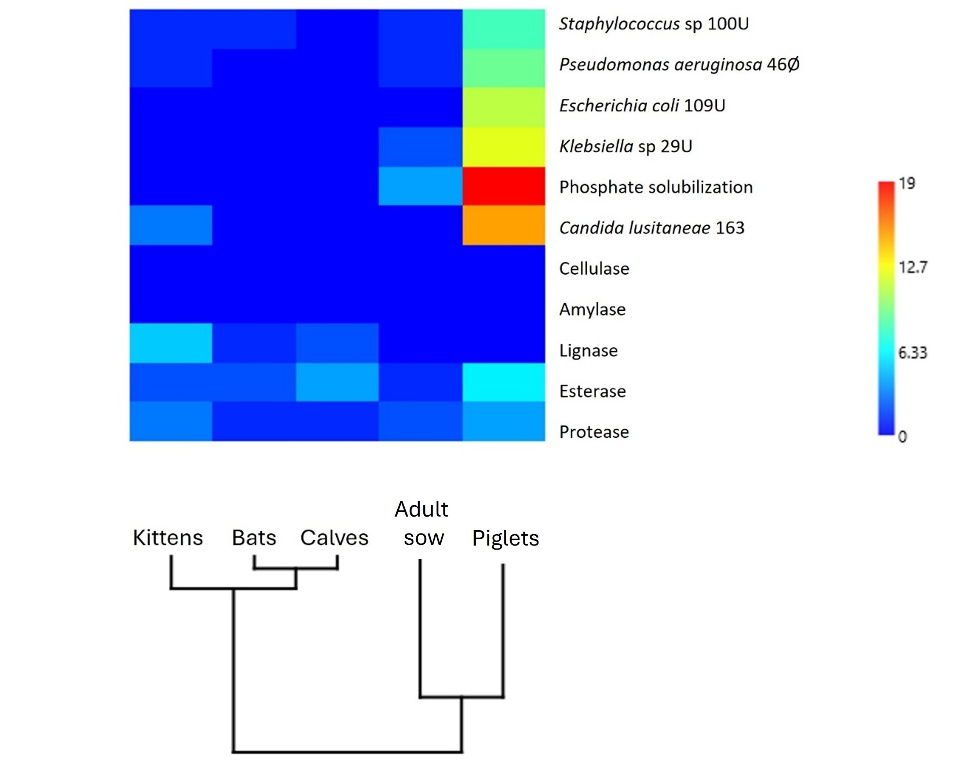


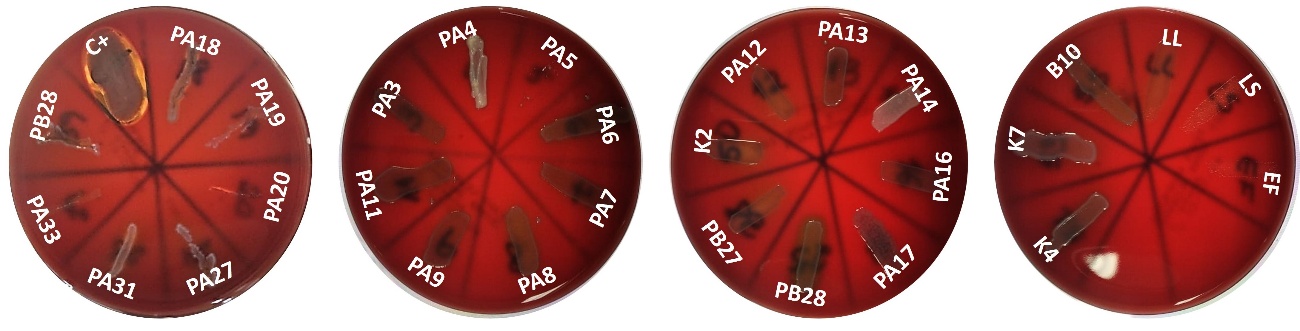

Supplement: Supplementary file 1 — Supplementary Material 1 (DOCX 265 KB) [file 11274_2026_4801_MOESM1_ESM.docx]
