# Supplementary figures and images for: Lactic acid bacteria isolated from mammalian feces exhibit distinct diversity and probiotic traits
Source: World J Microbiol Biotechnol. 2026 Mar 10;42(3):128. doi: 10.1007/s11274-026-04801-8 (PMC12971750; doi:10.1007/s11274-026-04801-8)

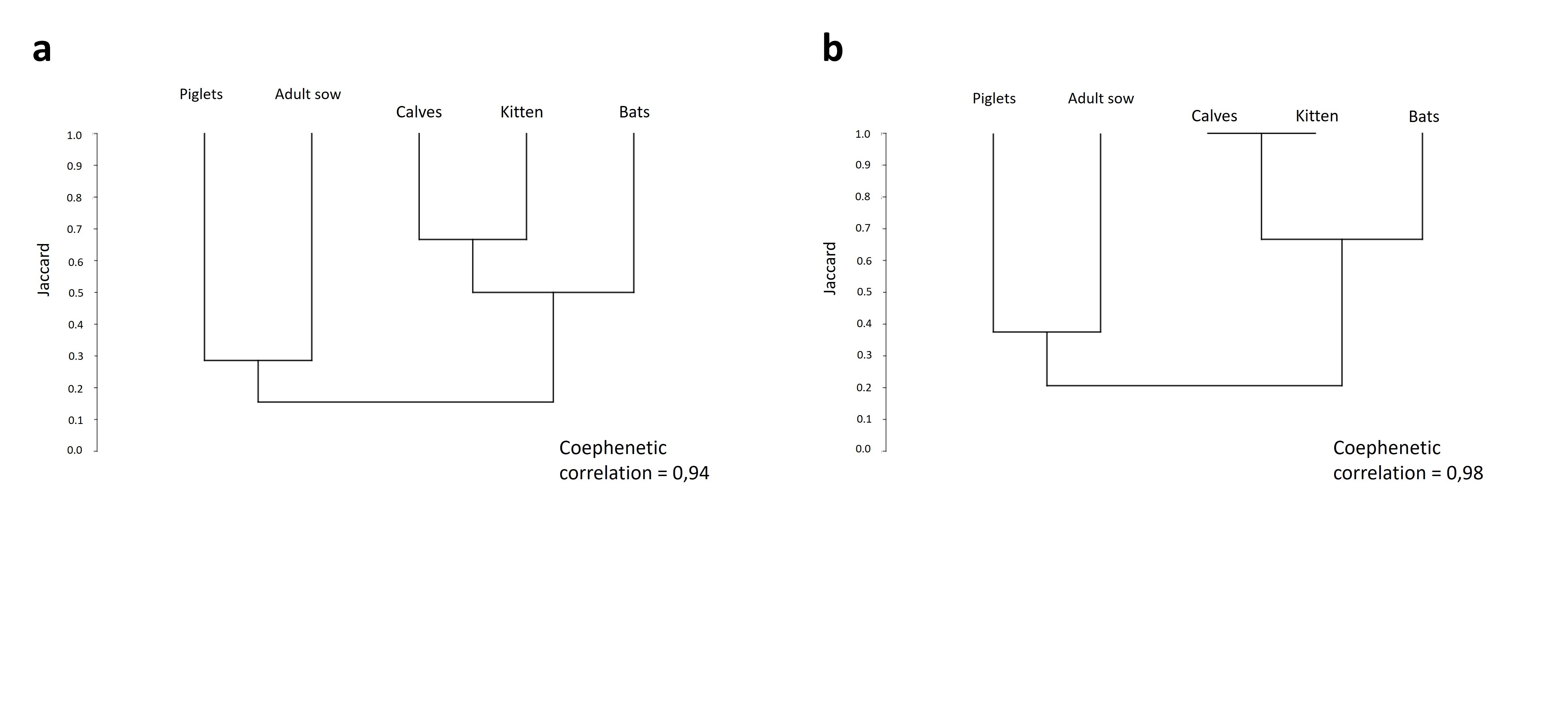

Supplement: Supplementary file 3 — Supplementary Material 3 (JPG 190 KB) [file 11274_2026_4801_MOESM3_ESM.jpg]

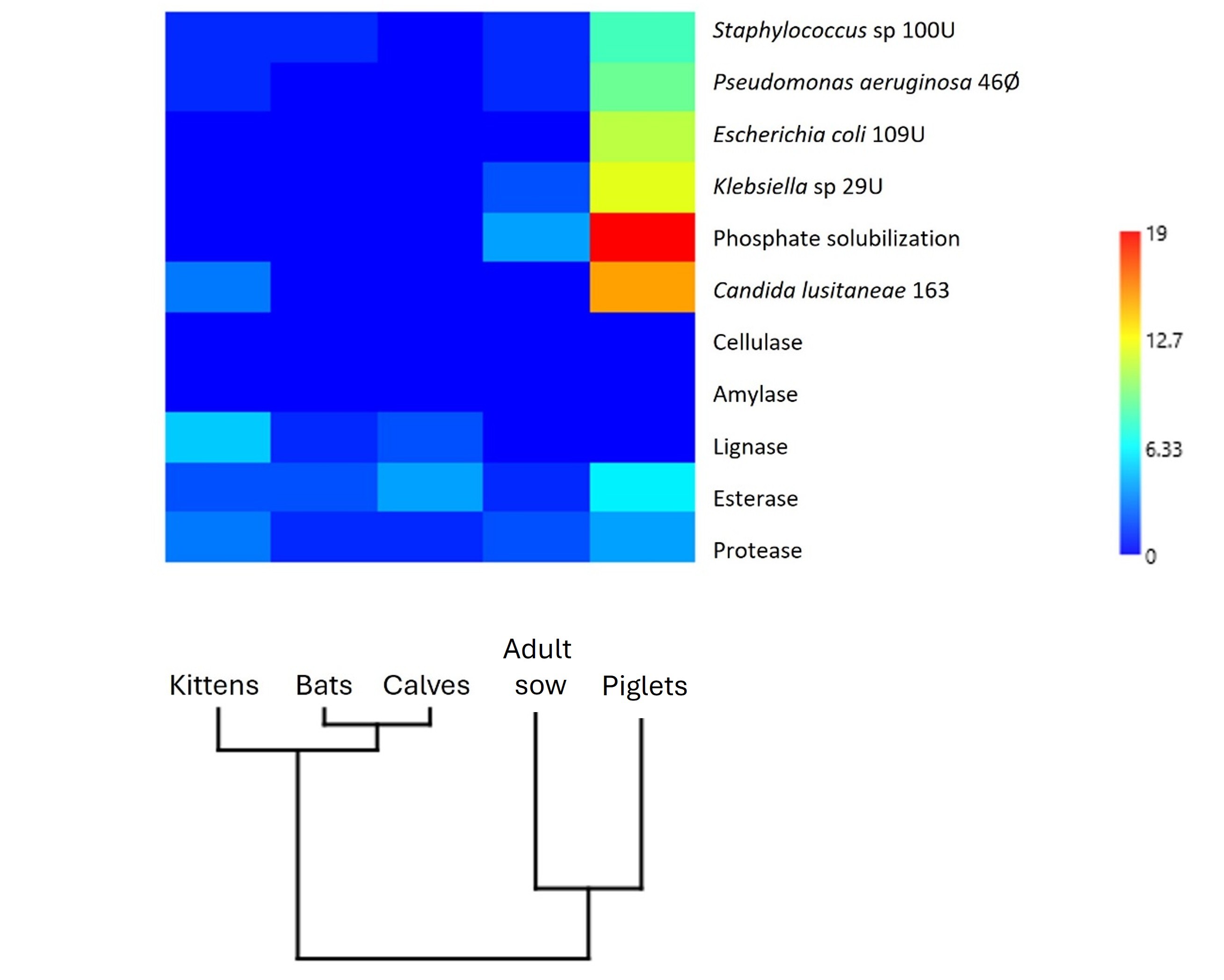

Supplement: Supplementary file 4 — Supplementary Material 4 (JPG 186 KB) [file 11274_2026_4801_MOESM4_ESM.jpg]

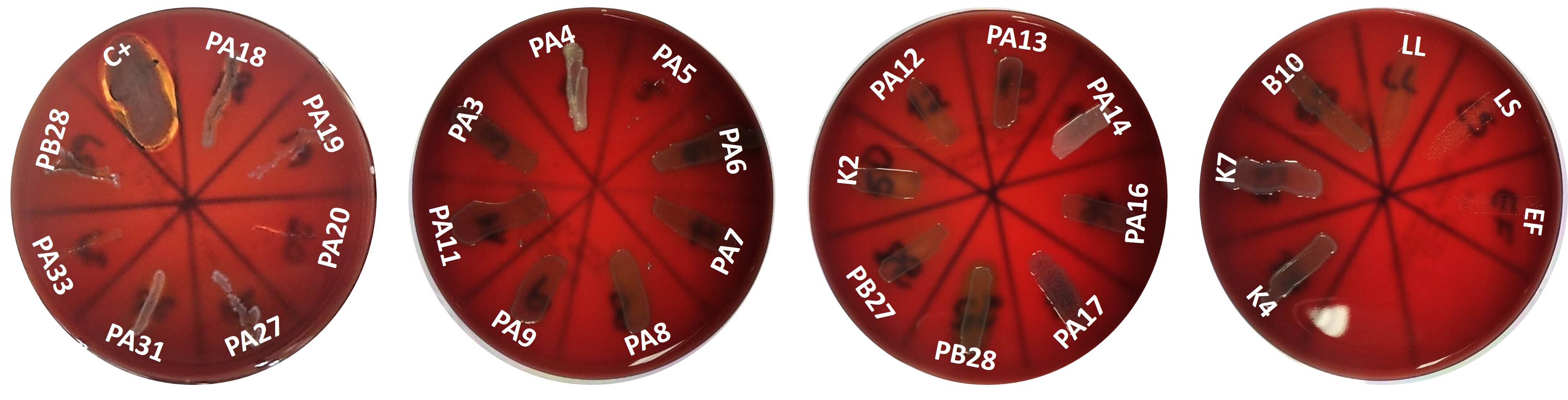

Supplement: Supplementary file 5 — Supplementary Material 5 (JPG 490 KB) [file 11274_2026_4801_MOESM5_ESM.jpg]
